# s‐scheme3D/3D Bi^0^/BiOBr/P Doped g‐C3 N4 with Oxygen Vacancies (Ov) for Photodegradation of Pharmaceuticals: In‐situ H_2_O_2_ Production and Plasmon Induced Stability

**DOI:** 10.1002/cssc.202401471

**Published:** 2024-10-18

**Authors:** Mope E. Malefane, Muthumuni Managa, Thabo T. I. Nkambule, Alex T. Kuvarega

**Affiliations:** ^1^ Institute for Nanotechnology and Water Sustainability, College of Science, Engineering and Technology University of South Africa Florida, Johannesburg 1709 South Africa

**Keywords:** Electron transfer, Levofloxacin and oxytetracycline, Photo-fenton, S-scheme, Surface plasmon resonance

## Abstract

Complications accompanying photocatalyst stability and recombination of exciton charges in pollutants degradation has been addressed through the construction of heterojunctions, especially S‐scheme heterojunction with strong and distinctive redox centres. Herein, an S‐scheme BiOBr (BOR) and g‐C_3_N_4_PO_4_ (CNPO) catalyst (BORCNPO) with oxygen vacancy (Ov) was synthesized for levofloxacin (LVX) and oxytetracycline (OTC) photodegradation under visible light. The 3D/3D BORCNPO catalyst possessed C−O−Br bridging bonds for efficient charge transfer during the fabrication of S‐scheme heterojunction. In‐situ H_2_O_2_ formation affirmed by potassium titanium (IV) oxalate spectrophotometric method improved the mineralization ability of BORCNPO7.5 catalyst. Bi^0^ surface plasmon resonance (SPR) enhanced formation and involvement of ⋅O_2_^−^ and the stability of the catalyst which increased reaction rate with increasing cycling experiments. XPS and radical trapping experiments supported the S‐scheme charge transfer mechanism formation with high degradation rate of LVX which was 3 times higher than OTC degradation rate. Mineralization of pollutants and their intermediates were demonstrated with florescence excitation and emission matrix (FEEM) and quadruple time of flight high performance liquid chromatography (QTOF‐HPLC). This work advances development of highly stable and efficient catalysts for photodegradation of pollutants through the formation of S‐scheme heterostructure.

## Graphical abstract

The current work investigates the formation of an S‐scheme heterojunction between two three dimensional (3D) semiconductors BiOBr (BOR) and P‐g‐C_3_N_4_ (CNPO) with oxygen vacancies and plasmonic Bi^0^ for enhanced stability, electron transfer, reactive oxygen species (ROSs) generation, and in‐situ H_2_O_2_ formation and decomposition to turn waste into wealth (OH⋅) towards enhance degradation of oxytetracycline and levofloxacin.

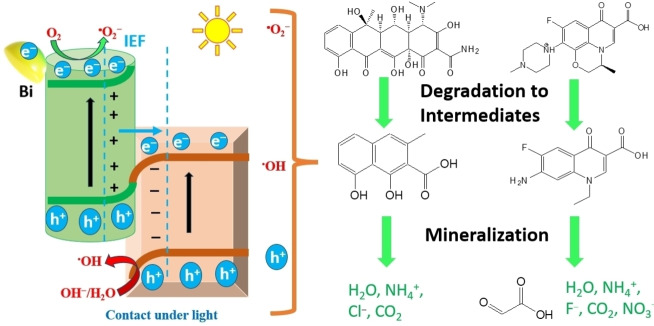

## Introduction

Pharmaceuticals invention and use has escalated in the past decade to counterattack the advent of new diseases and improve human well‐being, principally since the emergence of COVID‐19 in 2019. The increased consumption of pharmaceuticals is directly proportional to their discharged concentrations into the environment which pollutes the receiving environment, predominantly water bodies. Among the pollutants, oxytetracycline and levofloxacin are the most consumed for treatment of diseases caused by gram positive and gram‐negative bacteria due to their broad spectral antibacterial activity and they are the most detected pharmaceuticals in surface waters.[^1^, ^2^] Therefore, different groups have dedicated time to find innovative ways to completely degrade these pollutants into small and biodegradable molecules that are less hazardous than the parent compounds.

A series of methods were established for effective removal of hazardous pollutants due to their possible human toxicity and ecosystem deterioration with advanced oxidation processes (AOPs) pioneering studies in recent years.[^1^, ^3^, ^4^] Among AOPs, photocatalysis is recognized as a sustainable and effective method with potential for mineralization of organic pollutants to small and less toxic by‐products. Several semiconductors have been investigated for their potential in practical applications with a consensus reached that formation of heterojunctions is the best strategy for combating limitations of single semiconductors use such as high recombination of charge carriers, surface reactions, and low quantum efficiency.[^5^, ^6^] Based on band structure and pathway of electrons transfer, heterojunctions can be classified into different categories such as Z‐scheme, C‐scheme, type II, type I, S‐scheme, etc. with the recently discovered S‐scheme heterojunction the most prominent.[^7^, ^8^, ^9^] This is because the S‐scheme heterojunction fabrication was proposed with extensive investigation based on the recent knowledge and understanding of charge transfer principles.^[10]^ Therefore, fabrication of S‐scheme heterostructures has become increasingly popular.

S‐scheme heterojunction principle considers: (1) Presence of staggered band alignment where the conduction band (CB) of one semiconductor, oxidation photocatalyst (OP), is between the CB and the valence band (VB) of another, reduction photocatalyst (RP). (2) There is a formation of internal electric field (IEF) when the two semiconductors come into contact under dark conditions with electrons jumping from a semiconductor with small work function to the one with a larger work function.^[11]^ (3) Lastly, the induced IEF results in band bending which influences interfacial charge recombination for formation of distinctive redox sites maintaining the high redox potential of the system for efficient redox reactions. The high reduction and oxidation potentials of RP and OP are important for generation of reactive oxygen species (ROSs) like ^1^O_2_, H_2_O_2_, ⋅O_2_^−^, ⋅OH, and h^+^ that are involved in pollutants abatement.^[11]^ Therefore, S‐scheme heterojunction investigations are important for the creation of sustainable solutions towards degradation of pharmaceuticals.

Selection of semiconductors is an important component towards the design of efficient S‐scheme heterojunctions, and visible light active semiconductors offer a promising prospect towards efficient utilization of light in the broad solar spectral range which possess more visible and NIR light than UV light. Thereinto, g‐C_3_N_4_ offers great prospect of visible light absorption (E_g_=2.7 eV), Frenkel excitons resulting in localized electronic states and strong exciton binding energy (EBE), and less toxicity as a non‐metal semiconductor photocatalyst.^[12]^ However, it is unexceptional to recombination of photoexcited electrons and holes associated with the use of single semiconductors. Doping is approved as an imperative strategy for opposing the recombination of photoexciton pairs and modulation of the band structure of semiconductors with non‐metal dopants preferred in fabrication of composites for water treatment to minimize metal leaching.^[13]^ Interestingly, P dopants have been investigated on g‐C_3_N_4_ with results demonstrating the improvement in activity due to alteration of the band structure and long life‐time of electrons for stable and efficient ROSs formation.[^12^, ^14^] Therefore, doping g‐C_3_N_4_ with P was employed to form a semiconductor with a different band structure during construction of S‐scheme heterostructure. Moreover, literature proved that doping induced defects for improved charge separation and ROSs generation.^[14]^

In‐situ H_2_O_2_ generation during photocatalytic reactions has been determined as a green technology for manufacturing of H_2_O_2_ due to its high demand for applications such as fuel cell carriers, making of chemicals, sterilization, and environmental remediation.[^15^, ^16^] In environmental remediation, in‐situ H_2_O_2_ substitutes the need for its external addition that poses human health hazards due to its corrosive nature. The H_2_O_2_ formation depends on the band structure of the selected semiconductors and occurs through a two‐electron process (Equation 1).^[17]^ Moreover, its efficient utilization is important for generation of ROSs (especially ⋅OH) through different mechanisms such as photon or heat energy (Equation 2. This preferred process is often hindered by antagonist reactions for consumption of electrons (Equation 3) and holes (Equation 4) which retards the rate and efficiency of ROSs formation through exciton pairs.[^18^, ^19^] However, photon or heat activation process is energy intensive which limited the applicability of Equation (2). Except the heat activation, metals with redox cycles have demonstrated capabilities towards Fenton like reactions such as Fe, Mn, Ru, Ce, Co, Cu, etc.[^20^, ^21^] Therefore, the waste H_2_O_2_ generated in‐situ can be turned into ⋅OH wealth through Fenton‐like reactions for efficient degradation of pollutants. Moreover, vacancies or defects and carbon materials can act as activation centres for H_2_O_2._ ^[20]^

(1)

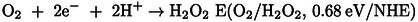



(2)

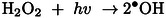



(3)

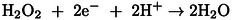



(4)

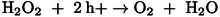




During heterojunctions development, bismuth‐based photocatalysts, particularly bismuth oxyhalides BiOX (X=F, Cl, Br, I) are of tremendous significance on account of visible light absorption capability, excellent prospect for pollutants degradation, and unique electronic structure.[^22^, ^23^] BiOBr is interesting because like other BiOX semiconductors, it has a PbFCl crystal structure with tetragonal arrangement that constitutes of alternating layers of halogen (Br) and [Bi_2_O_2_]^2+^ slab with a band gap of 2.8 eV.^[24]^ BiOBr is mostly synthesized with hydrothermal method which often results in the presence of metallic Bi that assists with its surface plasmon resonance for increased light harvesting capability across the solar spectrum.[^25^, ^26^, ^27^] Guan et al. reported plasmonic Bi/BiOBr catalyst coupled with carbon quantum dots for efficient light absorption and C‐scheme heterojunction construction through ohmic junction and SPR induced light absorption.^[28]^

BiOBr has demonstrated its ability to act as both a RP and OP based on the band structure of the semiconductor it is coupled with which makes it an interesting choice to be coupled with P doped g‐C_3_N_4_ which also acts as an OP and RP.[^25^, ^29^, ^30^] Additionally, defects such as oxygen vacancies are reported on BiOBr for enhanced photocatalytic degradation of organic pollutants.^[30]^ Just recently, a P doped g‐C_3_N_4_ heterojunction was reported by Feng et al. towards enhanced molecular oxygen activation for phenanthrene degradation with ⋅O_2_^−^, ⋅OH, and h^+^.^[31]^ Interestingly, a g‐C_3_N_4_ and BiOBr based heterojunction with Fe MOF as electron mediator was constructed for ciprofloxacin degradation.^[32]^ DFT calculations demonstrated that BiOBr was the RP while g‐C_3_N_4_ was the OP for ciprofloxacin degradation enhanced by presence of ⋅O_2_^−^ and h^+^. Therefore, fabrication of BiOBr and g‐C_3_N_4_ based S‐scheme constructures are fascinating and worth investigation for pollutants degradation.

Motivated by the above discussions, an S‐scheme based heterojunction (BORCNPO7.5) was successfully synthesized with OP as P doped g‐C_3_N_4_ (referred to as CNPO) and BiOBr (BOR) as RP for degradation of OTC and LVX. Worm‐like BOR was deposited on the surface of rock stone‐like CNPO forming a bridging bond for efficient charge transfer while plasmonic Bi metal enhanced the visible light absorption capacity of the composite and improved its stability as it increased with recycle runs. XPS analysis proved the S‐scheme heterojunction and radical trapping experiments affirmed the existence and involvement of ⋅O_2_^−^, ⋅OH, and h^+^. Moreover, in‐situ H_2_O_2_ formation and consumption improved the mineralization capability of LVX and OTC by the S‐Scheme BORCNPO7.5 as proved by QTOF‐HPLC‐MS. This work investigated the dual in‐situ H_2_O_2_ generation and consumption for pollutants degradation on a SPR Bi/BOR/CNPO catalyst with plasmonic induced stability for the first time.

## Methodology

### Materials

AgNO_3_ (≥99 %), Bi(NO_3_)_3_.5H_2_O (98 %), C_3_H_6_N_6_ (99 %), KBr (≥99 %), C_4_K_2_O_9_Ti.2H_2_O (≥99.0 %), C_6_H_4_O_2_ (BQ, ≥98 %), C_2_H_5_OH, (70 %), CH_3_OH (99.8 %), C_22_H_24_N_2_O_9_.HCl (OTC), C_3_H_8_O (IPA, >98 %), C_18_H_20_FN_3_O_4_ (LVX), and CH_3_(CH_2_)_11_OSO_3_Na (SDS, ≥98 %), CH_3_CO_2_H (GAA, ≥100 %), H(OCH_2_CH_2_)_n_OH (PEG600, MW=600), H_2_SO_4_ (95.0–98.0 %), KMnO_4_ (≥99 %), Na_2_HPO_4_ (≥99 %), NaH_2_PO_4_ (≥98 %), NaHCO_3_ (≥95 %), NaNO_3_ (≥99 %), NaCl (≥99 %), were used as purchased from Sigma‐Aldrich, South Africa. C_10_H_14_N_2_Na_2_O_8_⋅2H_2_O (EDTA‐2Na, 99.0–101.0 %) was purchased from SAARCHEM company, South Africa. Chemicals were used without further purification and ultrapure deionized water (Millipore 18.2 cm Ω) was used for making solutions.

### Synthesis of CNPO

The method included the mixing of 5 g melamine and 5 g uric acid with 1 g sodium adiabatic phosphate in a wt % ratio of 5 : 5 : 1. The precursors were grinded with pestle and mortar to a fine powder and subjected to calcination at 450 °C for 4 h. The obtained powder was centrifuge washed with ethanol and water 5 times to remove impurities and unreacted precursors and then vacuum dried at 50 °C for 10 h.

### Synthesis of Bi^0^/BiOBr/CNPO

**Figure 1:**
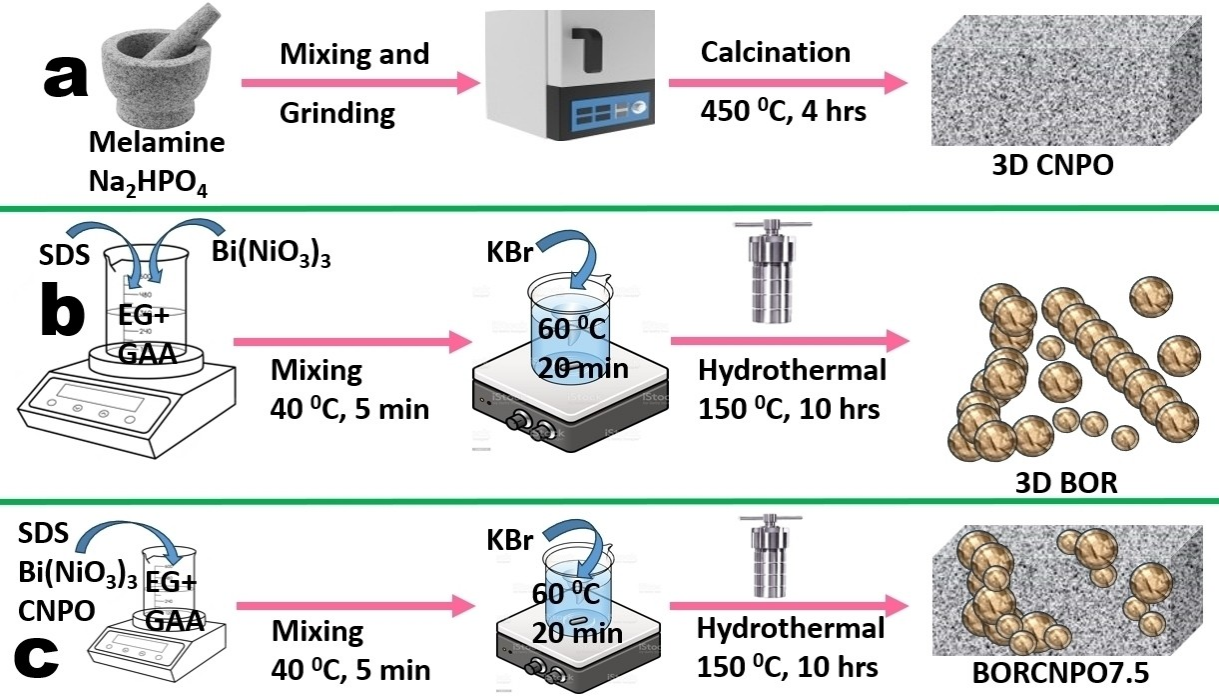
Synthesis of (a) CNPO, (b) BOR and (c) BORCNPO composites.

The synthesis of BiOBr was performed by mixing 0.416 g of SDS and 5.12 g of Bi(NO_3_)_3_.5H_2_O in a mixture of glacial acetic acid (75 ml) and equal volume of ethylene glycol, MW=600 (EG600) at 40 °C under vigorous stirring for 5 min. Then 1.12 g of KBr was added to this mixture and the temperature was adjusted to 60 °C. Stirring was continued for a further 20 min and the contents were transferred into a 200 ml Teflon lined steel autoclave to continue the reaction in an oven at 150 °C for 10 h. The sample was collected by centrifuge, washed three times with distilled water and ethanol before being dried in a vacuum oven at 50 °C for 12 h. It was then denoted as BOR and stored for further use. The synthesis of the composites was done following the same procedure for BiOBr synthesis with the addition of 0.5, 0.75 and 1 g of CNPO before addition of SDS to make composites labelled as BORCNPO5, BORPO7.5 and BORCNPO10 respectively. Using weight ratios in the final BORCNPO7.5 composite, a physical mixture of CNPO and BOR was made through grinding with a mortar and pestle for comparison. The synthesis of pristine semiconductors and the composite is presented in Figure 1.

### Characterization

Different characteristics were investigated using characterization tools to understand different properties of the material. Confocal WITec TS‐150 (molecular vibrational modes with 532 nm laser), PerkinElmer FTIR frontier 100 spectrometer (bond vibrations with KBr method), Rigaku SmartLab X‐ray diffractometer equipped with a Cu Kα X‐ray source at wavelength of 1.5418 Å and a current and voltage of 200 mA and 45 kV (crystal structure at 2θ range of 10° to 70°), Zeiss Field Emission Scanning Electron Microscope (FE‐SEM) coupled with an Oxford EDX detector and JEOL JEM 2100 at 200KV (surface and internal morphology), Thermo ESCAlab 250Xi spectrometer with Al kα (1486.7 eV) (surface chemical composition analysis with X‐ray power of 300 W), PerkinElmer UV‐Vis Lambda 650S spectrometer using BaSO_4_ as reference (light absorption properties at a range between 200–800 nm), and Horiba Fluorog‐2 Jobnyvon spectrometer at 325 nm excitation wavelength (fluorescence emission spectra). Electrochemical properties were obtained on a Bio‐logic SAS SP‐50e electrochemical workstation with a three electrode configuration using reference (Ag/AgCl (3 M KCl), counter electrode (Pt wire), working electrode (sample drop cast on FTO glass), and 0.1 mM Na_2_SO_4_ as electrolyte. The sample (9 mg) was dropcasted on the working electrode with PVA (1 mg) as binder in dimethyl sulfoxide DMSO (1 ml) solution. UV‐Vis Lambda 650S spectrometer, Aqualog HORIBA Jobin Yvon fluorescence spectrometer, and QTOF‐HPLC‐MS were used to follow degradation of pollutants to obtain absorbance, 3D Fluorescence Excitation Emission Matrix FEEM and intermediates by‐products, respectively.

### Photodegradation Experiments

Photodegradation experiments were conducted on a custom‐made cuboidal photoreactor with installed LED lights on opposite sides consisting of 9 LEDs on each of two opposite sides making a total of 18 LED lights of 5 W each making a total of 90 W (λ≥425 nm) with intensity of 12 mWcm^−2^ as measured by a digital lux meter. The light intensity was set at 50 %. In a basic experiment, 10 mg catalyst was added to 100 ml of 10 ppm LVX or OTC and stirred for 30 min in the dark for optimum pollutant and catalyst interaction. The unadjusted pH of the solution was 6.32. Lights were turned on and sampling was done at 10 min intervals and filtered through 0.45 μm PVDF membrane filters. The degradation of pollutants was followed with UV‐Vis by measuring absorbance between 500–200 nm for OTC and from 400–200 nm for LVX. Other pollutants that were tested include diclofenac (DFC), sulfamethoxazole (SMX), and bisphenol A (BPA). The degradation efficiency (η) and rate constants (K_obs_, min^−1^) were evaluated using Equations (5) and 6.

(5) $$\eta \%=\;\frac{C_{0}-C_{t}}{C_{0}}\;X\;100\;\%\;$$

(6) $$\ln\left(\frac{C_{t}}{C_{0}}\right)=\;-K_{obs}t\;$$

where *C_0_ and C_t_* is the initial (0) and final (t) concentration of pollutant at time (t) during the reaction. The degradation by‐products were also analyzed with FEEM and HPLC‐MS to affirm degradation and pathway. The experiments were carried out in triplicates under similar reaction conditions. The global degradation efficiency (GDE) of the pollutants were estimated by ($\frac{{\%DE}_{OTC}+\;{\%DE}_{LVX}+{\%DE}_{DFC}+{\%DE}_{SMX}+{\%DE}_{BPA}}{5}$ ).^[33]^ Moreover, reuse experiments were performed to investigate the stability of BORCNPO7.5 composite under similar conditions used for LVX and OTC photodegradation. After the reaction was completed, the catalyst was washed, centrifuge recovered and dried in an oven at 80 °C for 8 h. The cycles were repeated 5 times, and the catalyst demonstrated colour change from light grey to light brown from the third cycle of the five cycles. The degradation of OTC and LVX was monitored in the presence of radical scavengers. Addition of 10 mM of EDTA, IPA, and BQ as scavengers for h^+^, OH⋅, and ⋅O_2_^−^ to determine the involvement and presence of these reactive oxygen species (ROSs) during degradation of pollutants.

### H_2_O_2_ Production

The presence and consumption of H_2_O_2_ was investigated through potassium titanium (IV) oxalate spectrophotometric method. In a simple experiment, 10 mg catalyst was dispersed in 100 ml of deionized water, and adsorption‐desorption equilibrium was allowed for 30 min. Aliquots of 1 ml were withdrawn from the reactor at stipulated time intervals and filtered to remove residual catalyst. Then 1 ml of 0.1 mM C_4_K_2_O_9_Ti.2H_2_O was added and mixed well before the mixture was analyzed using UV‐Vis at a wavelength of 400 nm to determine the concentration of H_2_O_2_ from the calibration curve presented in Figure S1a. The H_2_O_2_ decomposition was investigated by adding 10 mg of catalyst in 50 μM of H_2_O_2_ solution. Before irradiation, the catalyst was stirred in the dark for 30 min and then the residual H_2_O_2_ concentration was measured. Trapping experiments were performed to detect the ROSs that influence H_2_O_2_ production by adding BQ, and HCOOOH (10 %) as scavengers for superoxide, electrons, and holes.

## Results and Discussion

### Characterization of Composites

**Figure 2:**
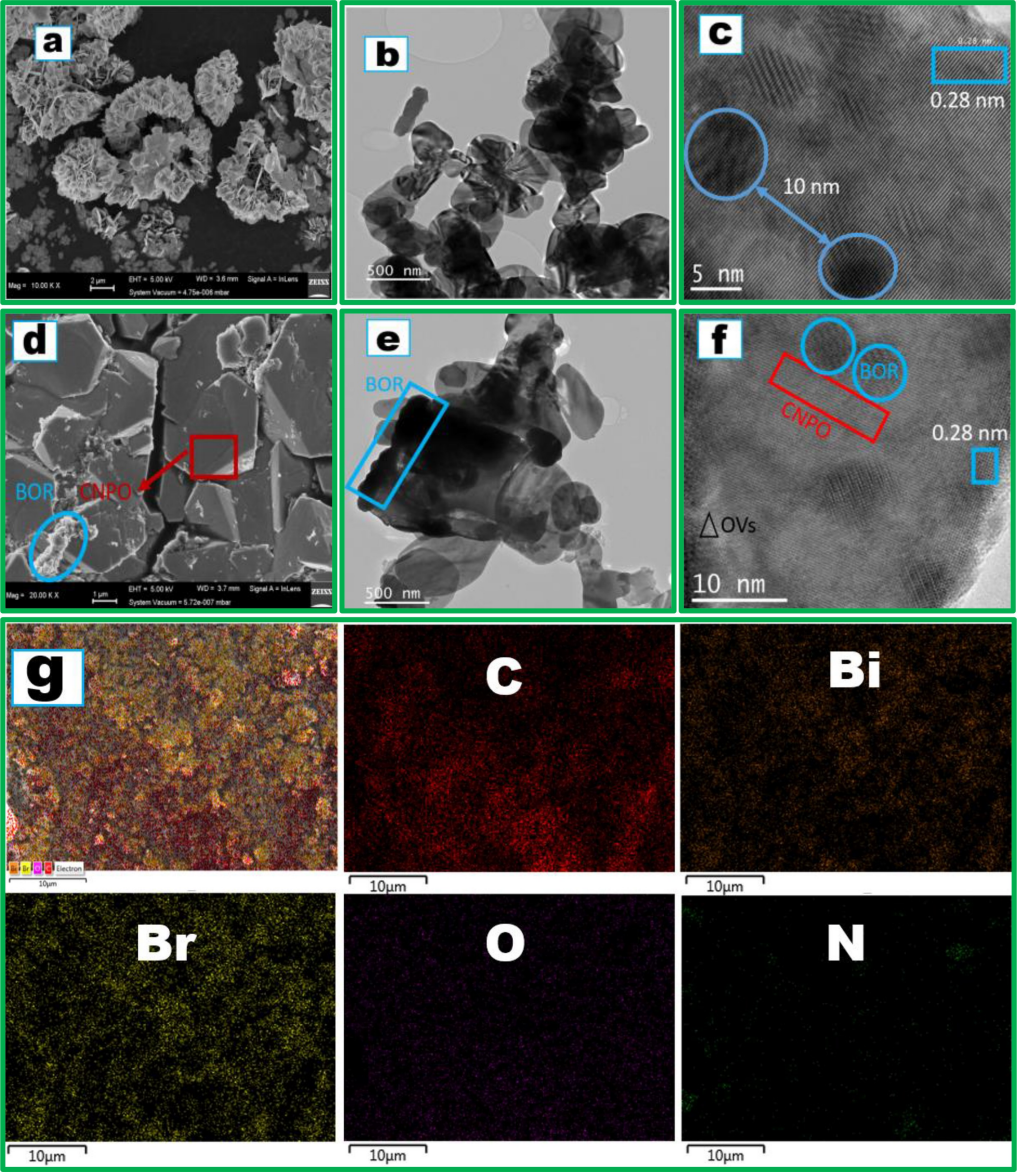
BOR (a) SEM, (b) TEM and (c) HR‐TEM. (d) SEM, (e) TEM, (f) HR‐TEM and (g) EDX mapping of BORCNPO7.5.

Figure 2a presents the scanning electron microscopy (SEM) image of BiOBr revealing platelets that are arranged into spherical microspheres that inter‐connected and grew into rods. In Figure 2b, TEM affirmed the observed morphology with SEM and showed that discs of BOR plates aggregated into spherical shapes and confirmed that the observed rods were caused by aggregation of the discs. Figure 2c demonstrated the existence of 0.28 nm fringes of BOR indexed to its [102] facets^[28]^ which further supported that the distance between the microspheres that formed a rod was around 10 nm. When BORCNPO7.5 composite was fabricated, the CNPO 3D stone rock‐like with rough surfaces formed with deposition of aggregated microspheres that form worm‐like rods in Figure 2d. The formed 3D worm‐like microspheres had different sizes and were observed on the surface of CNPO 3D rocks. The cracks are important for improved interaction of the photocatalyst with pollutant for efficient degradation. The 3D stone rock‐like CNPO had cracks for pollutant interaction and surface roughness linked with high photocatalytic activity.^[31]^ The worm‐like segments were made of spherical aggregation of BOR plates which affirmed that the BOR morphology was unchanged during the heterojunction fabrication. Moreover, the worm‐like rods interconnected different plates of CNPO as stipulated by TEM in Figure 2e. This could have ensured strong contact between the CNPO and BOR during the proposed heterojunction fabrication. The results suggested that CNPO and BOR could interact which would result in possible formation of heterojunction. Figure 2f demonstrated the existence of rocky CNPO strongly interacting with microspheres of BOR with 5 nm or less between them. The proposed formation of a heterojunction decreased the distance between the BOR microspheres deposited on CNPO which would suggest that the heterostructure formation probability increased. BOR did not change its crystalline facets, and more oxygen vacancies were observed in the composite. The results supported the proposed S‐scheme heterojunction formation with EDX mapping supporting the existence of all expected elements except P which could be due to low amount which reduced when CNPO was doped with BOR (Figure 2g). The mapping of BOR only showed Bi, Br and O elements as depicted in Figure S1b which demonstrated purity of the synthesized materials. In Figure S2a, EDX spectra of BOR affirmed all expected elements with O having the lowest weight percentage which could signal oxygen deficiency. EDX spectra of BORCNPO7.5 depicted the presence of all expected elements of Bi, Br, C, N, P, and O with O less than Br which could be due to oxygen vacancies as depicted by Figure S2b. Therefore, a heterojunction would fruitfully be assembled with BOR and CNPO which was proposed as S‐scheme.

**Figure 3:**
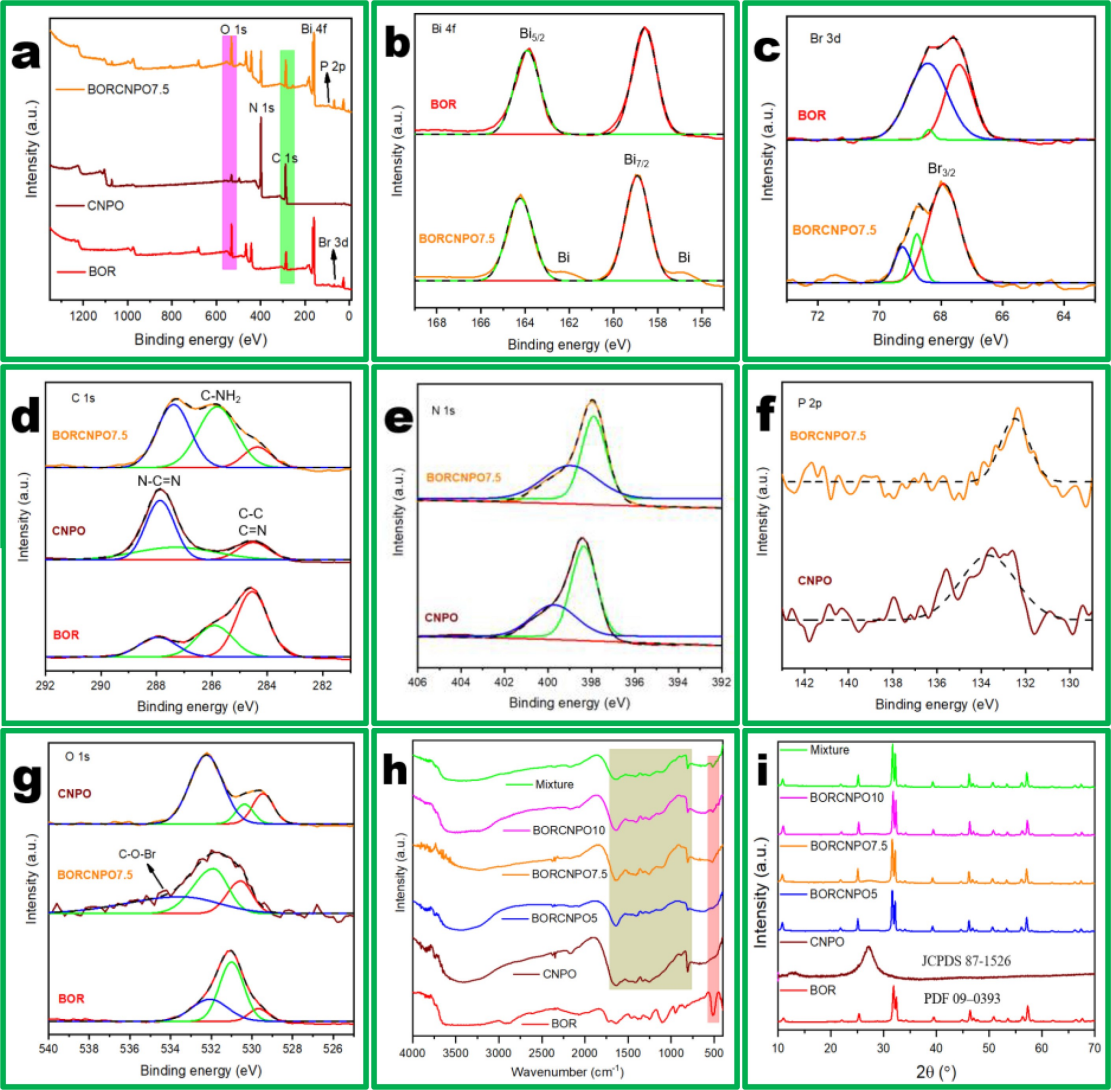
BOR, CNPO, and BORCNPO (a) XPS survey, (b) Bi 4 f, (c) Br 3 d, (d) C 1s, (e) N, (f) P 2p, and (g) O 1s spectra. (h) FTIR and (i) XRD of synthesized composites.

XPS was used to understand the chemical properties and electronic state of the as‐prepared BOR, CNPO, and BORCNPO7.5. Figure 3a demonstrates that all expected elements of Bi, Br, N, C, P, and O were observed on the full survey spectra of CNPO, BOR, and BORCNPO7.5 which supported EDX results. The high resolution XPS spectra was employed to understand the oxidation states, heterojunction formation and to understand the possible mechanism of charge transfer. The Bi 4 f spectra for BOR and BORCNPO7.5 had doublet peaks of Bi 4f_5/2_ at 163.88/164.25 eV and Bi 4f_7/2_ at 159.1/158.9 eV, respectively (Figure 3b).^[31]^ Moreover, the Bi 4 f spectra of BORCNPO7.5 had two new peak at 156.87 and 162.22 eV which is inclined to the presence of plasmonic Bi.^[28]^ Therefore, the formed heterostructure had plasmonic effect from Bi which improved the light absorption capacity.

Additionally, the Br 3d peak of BOR and BORCNPO appeared at binding energies of 67.42/67.89 eV and 68.45/68.78 eV corresponding with Br 3d_5/2_ and Br 3d_3/2_ as shown in Figure 3c.^[34]^ In the composite, a new peak appeared at a binding energy of 71.43 which was corresponded to C−O−Br bridge. This could imply that the interaction between the proposed heterojunction occurred through the formation of an electrophilic C−O−Br bridging bond. To further confirm this proposal, the C 1s spectra is investigated as presented in Figure 3d. The C 1s peaks demonstrated the occurrence of C−C or C=N (284.46 eV), C−NH_2_ (287.07 eV), and N−C=N (287.89 eV) in CNPO while the same peaks in BORCNPO appeared at 284.23, 285.79, and 287.38 eV, respectively. The C 1s spectra of BORCNPO7.5 had a weak peak at binding energy (BE) of 291.32 eV which was indexed to the C−O−Br bridging bond. This corroborated the proposed covalent bond formation between C, O, and Br at the interface of CNPO and BOR. This would enhance strong interaction of the semiconductors in the heterojunction which would result in efficient interfacial charge transfer.

Figure 3e shows the N 1s spectra with BE of 398.33 and 399.75 eV in CNPO which were exhibited at BE of 397.89 and 399.13 eV in BORCNPO7.5 and corresponded well with pyridinic N and pyrrolic N in the carbon matrix, respectively. The absence of the C−N−H bond or oxidized N which is normally observed at around 400 eV^[31]^ could be indexed to substitution of N with P in the structure resulting in the reduction of terminal C−N−H bonds. Additionally, the P 2p peak in Figure 3f appeared at BE of 133.37 and 132.44 eV in CNPO and BORCNPO7.5, respectively. This weak peak accorded the observed low weight percentage of P which affirmed doping P into g‐C_3_N_4_. The O 1s spectra showed three peaks that are indexed to metal oxygen bond (M−O), OV defects, and surface adsorbed water (−OH/H_2_O).^[35]^ From Figure 3g, the peaks and their BE for BOR (530.52, 531.88, and 533.44 eV), CNPO (529.46, 530.39 and 532.19 eV) and BORCNPO7.5 (530.44, 531.71, and eV) corresponded with surface −OH/H_2_O, OV defects, and lattice oxygen of Bi−O, respectively. CNPO showed the smallest peak of OV defects which was very high in BOR and increased further in BORCNPO7.5. In addition, BORCNPO7.5 exhibited a new weak peak at BE of 534.26 eV which indicated the proposed C−O−Br bridging bond. The results supported interaction of CNPO and BOR at the interface through covalent bond formation which was further in solidarity with observed changes in BE.

To further understand the structure and functional groups of the nanomaterials, FTIR was used. For CNPO (Figure 3h), the N−H stretching vibrations and OH vibrations due uncondensed amino groups and surface‐adsorbed H_2_O, CN stretching modes, triazine units, and P−N stretching mode appeared at 3000–3800 cm^−1^, 1100–1650 cm^−1^, 795 cm^−1^, and 663 cm^−1^, respectively. The peak at 828 cm^−1^ corresponded well with C−H in plane bending while HCO_3_^−^ or KBr stretching vibration appeared at 2365 cm^−1^ and the N−H stretching of aromatic amines appeared at 2165 cm^−1^.^[36]^ The characteristic P peak was not observed due to the low content and overlap with C−N as reported in literature.^[37]^ BOR O−H, Bi−Bi, and Bi−O vibrations were observed at 1689 cm^−1^, 1234 cm^−1^, and 509 cm^−1^, respectively.^[31]^ In the composites, all the CNPO and BOR characteristic peaks appeared and demonstrated the successful fabrication of the composite with changes in triazine peaks suggesting interaction of the ring with BOR. This supported the formation of the bridging bond with the observed disappearance of the peak at 828 cm^−1^. This could be due to the structural defects on the triazine rings due to the proposed bridging bond formation.

XRD spectra is shown in Figure 3i. For CNPO, tris‐triazine [001] and graphite‐like structure stacking of [002] planes were observed at 2θ values of 12.9° and 27.1°, respectively^[31]^ which corresponded to JCPDS 87–1526^[38]^ while BOR peaks (PDF 09–0393) denoted the presence of the pure tetragonal phase.^[31]^ The wide [001] plane which is affirmed to induce OV defects was observed and it increased in intensity during fabrication of composites. This would imply that the composite has a higher probability for formation of more OV defects in support of HR‐TEM, EDX, and XPS analysis. Moreover, the composite showed the existence of only tetragonal BOR peaks with no other new peaks formed. In addition, no obvious diffraction peaks due to CNPO were observed in the composites but the presence of the lattice peaks due to CNPO were observed in Figure 2f which proved the presence of CNPO during fabrication of the composites.

### Degradation of Pharmaceuticals

**Figure 4:**
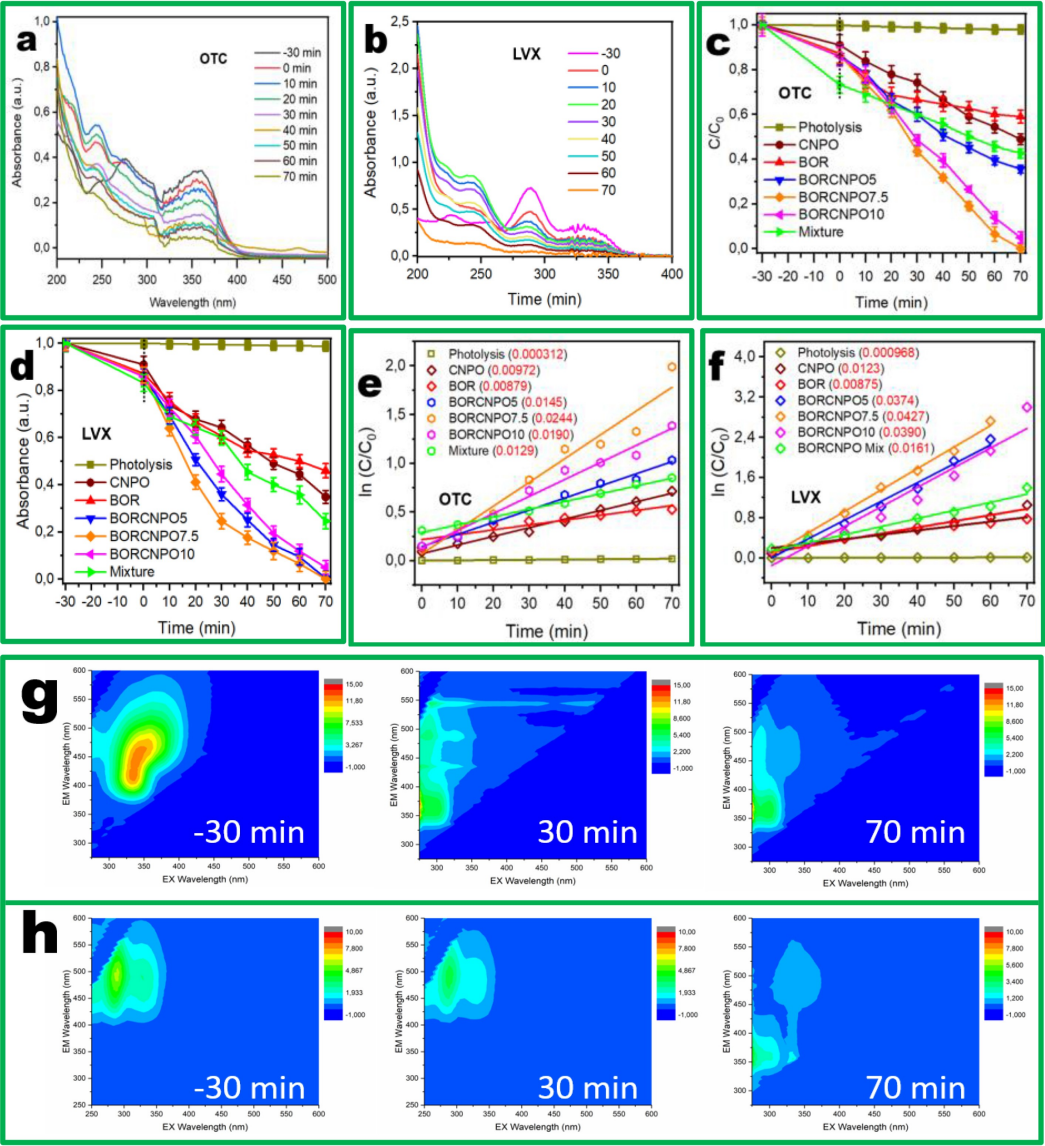
(a, b) UV‐Vis spectra, (c, d) photocatalytic degradation curves, (e, f) corresponding pseudo‐first‐order reaction kinetic curves with apparent rate constants k, and (g, h) fluorescence excitation emission matrix of OTC and LVX. Conditions: pH is 6.32, catalyst amount of 10 mg, and 100 ml solution of 10 ppm.

The adsorption equilibrium for the degradation of levofloxacin and oxytetracycline was investigated to occur within 30 min as shown in Figure S3a. Adsorption has negligible difference towards OTC and LVX removal. The UV‐Vis absorbance spectra during the degradation of OTC and LVX affirmed the reduction in absorbance during light irradiation with time. All three peaks of OTC decreased with time and after 40 min under light with BORCNPO7.5, a new peak appeared between 450–500 nm and decreased completely after 70 min (Figure 4a). In Figure 4b, LVX showed four peaks that decreased to a flat line after 70 min under light with BORCNPO7.5. Figure 4c showed that OTC photodegradation is negligible while CNPO, BOR, BORCNPO5, BORCNPO7.5, BORCNPO10, and physical mixture of CNPO and BOR demonstrated decrease in concentration with irradiation time. The degradation of OTC with mixture was higher than both CNPO and BOR and could demonstrate the additive photocatalytic degradation effect. CNPO gave the highest degradation rate than BOR and different compositions of BORCNPO gave the highest degradation rate. BORCNPO5 showed the smallest degradation rate than BORCNPO10 while BORCNPO7.5 gave the highest degradation rate for OTC removal.

The photodegradation rate of LVX with photolysis, CNPO, BOR and mixture followed a similar pattern to that of OTC. BORCNPO5, BORCNPO7.5, and BORCNPO10 gave comparable final degradation efficiency for LVX, and almost similar degradation rates as shown by Figure 4d. BORCNPO10 gave the smallest removal efficacy followed by BORCNPO5 which was followed by BORCNPO7.5 affirming that BORCNPO7.5 was the quickest catalyst for LVX degradation. The degradation data was fitted with first order reaction rate. Figure 4e–f showed the K_obs_ value of BORCNPO7.5 was 0.0244 min^−1^ and 0.0427 min^−1^ for OTC and LVX degradation, respectively. For all composites, LVX degradation rates were higher than OTC degradation rates. This could be better understood by chemical structures and the surface charges of OTC and LVX compared to the surface charge of BORCNPO7.5 which influences their interaction. For BORCNPO7.5, LVX degradation rate was 2.6 times higher than OTC degradation rate. Moreover, the FEEM spectra of OTC (Figure 4g) and LVX (Figure 4h) proved that OTC and LVX matrices changed with irradiation time affirming the degradation of these pharmaceuticals through formation and degradation of by‐products. These findings exhibited that BORCNPO7.5 has exceptional degradation performance for OTC and LVX with high TOC mineralization efficiency of 69.8 and 77.2 % in 70 min (Figure S3b), respectively.

The degradation of BORCNPO7.5 was tested against other pollutants. Bisphenol A (BPA), diclofenac (DFC), and sulfamethoxazole (SMX) were selected as other pollutants degraded with BORCNPO7.5 under light for 70 min. Figure S3c shows that their degradation followed the order of DFC>SMX> BPA. The first order degradation rate constants for DFC, SMX, and BPA were 0.0719, 0.0328, and 0.0221 min^−1^, respectively as demonstrated by Figure S3d. The Pearsons R‐square values were above 0.94 and they demonstrated a good correlation of the data. The global degradation efficiency (GDE) for all 5 pollutants gave 94.8 % which demonstrated that the BORCNPO7.5 composite has a high degradation efficiency for different pollutants. Therefore, this catalyst has potential for environmental pollution remediation.

To further investigate the merits of the synthesized BORCNPO7.5 catalyst, g‐C_3_N_4_ based catalysts were compared with this work by different reaction conditions (i. e. catalyst and pollutant concentration, source of light, degradation time, and solution pH), reaction rate, TOC (%) removal, the kind of pollutant, and the specific ROSs. Table 1 shows the current work degradation rate was higher than most similar works in literature and high mineralization efficiency was achieved with the LVX degradation the quickest and the one with high TOC mineralization efficiency than OTC degradation mineralization. Moreover, ⋅O_2_^−^ are the most involved ROSs on the BiOBr and g‐C_3_N_4_ based semiconductors that are compared in this work.

**Table 1:** Comparison of recent high impact work using similar catalysts towards degradation of pharmaceuticals.

Catalyst	Conditions	Rate (min^−1^)	TOC removal (%)	Pollutant	Involved ROSs	Ref
S‐scheme Bi/BiOBr/P‐doped g‐C_3_N_4_	[LVX]=10 mg/L, [cat]=0.1 g/L, 90 W LED lamps, pH=6.32, t=70 min	0.0427	77.2	Levofloxacin (LVX)	⋅O_2_^−^>⋅OH>h^+^	This work
S‐scheme Bi/BiOBr/P‐doped g‐C_3_N_4_	[OTC]=10 mg/L, [cat]=0.1 g/L, 90 W LED lamps, pH=6.32, t=70 min	0.0244	69.8	Oxytetracycline (OTC)	⋅O_2_^−^>⋅OH>h^+^	This work
MoSe_2_/Bi@BiOBr‐OV	[CTC]=10 mg/L, [cat]=0.6 g/L, visible light (λ>400 nm), pH=6.15, t=120 min	N/A	71.2	Chlortetracycline (CTC)	⋅O_2_^−^>⋅OH>h^+^	[39]
HDMP/CN	[OTC]=20 mg/L, [cat]=0.3 g/L, 300 W xenon lamp (λ>420 nm), pH=N/A, t=60 min	0.0290	15.4	Oxytetracycline (OTC)	⋅O_2_^−^>h^+^>⋅OH	[40]
g‐C3 N4/BiOBr S‐scheme	[TC⋅HCL]=10 mg/L, [cat]=0.5 g/L, 350 W xenon lamp, pH=6, t=60 min	0.0424	93.4	Tetracycline hydrochloride (TC⋅HCl)	⋅O_2_^−^>h^+^>⋅OH	[41]
Z‐scheme g‐CN/BiOBr/Fe_3_O_4_	[TC]=15 mg/L, [cat]=0.5 g/L, 300 W halogen lamp, pH=N/A, t=60 min	N/A	78.0	Tetracycline (TC)	h^+^>⋅O_2_^−^>⋅OH	[42]
S‐scheme P‐doped g‐C_3_N_4_/BiOBr	[PHE]=1 mg/L, [cat]=0.01 g/L, visible light, pH=7, t=90 min	0.0405	68.3	Phenanthrene (PHE)	h^+^>⋅OH>⋅O_2_^−−^	[31]
Z‐scheme Fe‐MOF@BiOBr/M^−^CN	[CIP]=10 mg/L, [cat]=0.63 g/L, 300 W xenon lamp (λ>420 nm), pH=N/A, t=160 min	0.0165	N/A	Ciprofloxacin (CIP)	h^+^>⋅O_2_^−^>e^−^>⋅OH	[32]
Z‐scheme CdSe/Se/BiOBr	[CIP]=20 mg/L, [cat]=1 g/L, 5 W LED, pH=2, t=30 min	0.112	79.5	Ciprofloxacin (CIP)	⋅O_2_^−^>⋅OH>h^+^	[22]
Sm_6_WO_12_/g‐C_3_N_4_ type II heterojunction	[LVX]=10 mg/L, [cat]=0.5 g/L, tungsten lamp (λ>420 nm), pH=N/A, t=70 min	0.034	N/A	Levofloxacin (LVX)	⋅O_2_^−^>⋅OH	[43]
S‐scheme Co_3_O_4_/Bi_2_MoO_6_@g‐C_3_N_4_	[LVX]=25 mg/L, [cat]=1.0 g/L, 300 W xenon, pH=4.45, t=60 min	0.0133	N/A	Levofloxacin (LVX)	⋅OH>⋅O_2_^−^>h^+^	[44]

### Adaptability and Stability

Adaptability of the catalyst was tested with different anions (H_2_PO_4_^−^, HCO_3_^−^, NO_3_^−^, and Cl^−^) that are inevitably detected in real environmental water samples.^[45]^ 10 mM concentrations of anions were added in the LVX reaction mixture with high degradation rate before dark experiments to understand their interaction during adsorption. Figure S3e showed that Cl^−^ did not affect the interaction between LVX and BORCNPO7.5, NO_3_^−^ sparingly reduced their interaction, while H_2_PO_4_^−^ and HCO_3_^−^ considerably reduced their interaction. The adsorption reduction observed with NO_3_^−^, H_2_PO_4_^−^ and HCO_3_^−^ implied presence of competitive mechanisms during adsorption of pollutant in their presence. NO_3_^−^ is reported to easily adsorb on the catalyst surface under acidic pH which would compete with negatively charged pollutants on the catalyst active sites and reduced their interaction.^[46]^ HCO_3_^−^ exists at pH>6.3 which also demonstrated its negatively charged and could inhibit pollutant interaction with catalyst while H_2_PO_4_^−^ can complex metallic species and retards their interaction with pollutants through (Bi^3+^+H_2_PO_4_^−^→BiH_2_PO_4_^2+^).^[46]^ Therefore, one mechanism of LVX degradation inhibition with NO_3_^−^, H_2_PO_4_^−^ and HCO_3_^−^ was through reduction of active sites due to physical adsorption while Cl^−^ had no effect on LVX degradation.

During the degradation process, Cl^−^ presence had negligible influence. The effect of Cl^−^ is related to its concentration, solution pH, and kind of pollutant. The low concentration of Cl^−^ would be indexed to the observed effect on LVX degradation since at lower concentrations, its scavenging reaction favours the reverse reaction to form ⋅OH (Equation (7)).^[46]^ NO_3_^−^ have higher scavenging effect on ⋅OH (E^0^=2.70 V) under acidic and near neutral pH conditions to produce NO_3_⋅ (E^0^=2.30 V) with low oxidation capacity (Equation (8)).^[47]^ Furthermore, HCO_3_^−^ scavenging of ROSs has also been documented in literature to occur through changes in solution pH to alkaline (as Equation (9) equilibrium shifts to the right) which decreases the oxidation potential of ⋅OH from E^0^=2.70 V to 1.5 V or direct reaction with H_2_O_2_ (HCO_3_^−^+H_2_O_2_→HCO_4_^−^+H_2_O) resulting in low oxidation capacity product as E^0^ (HCO_4_^−^/HCO_3_^−^)=1.8 V vs NHE.^[46]^ Lastly, H_2_PO_4_^−^ affected LVX degradation through changing the solution pH like HCO_3_^−^ which affected oxidative potential of ⋅OH, complexation with metal species that affects their involvement in H_2_O_2_ decomposition and scavenging of generated ROSs (Equation (10)).^[48]^ Therefore, effect of anions followed the order H_2_PO_4_^−^>HCO_3_^−^>NO_3_^−^ during LVX degradation while Cl^−^ showed negligible effect.
(7)

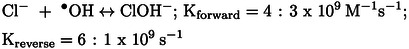



(8)

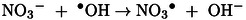



(9)

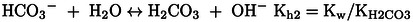



(10)

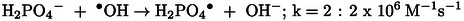




**Figure 5:**
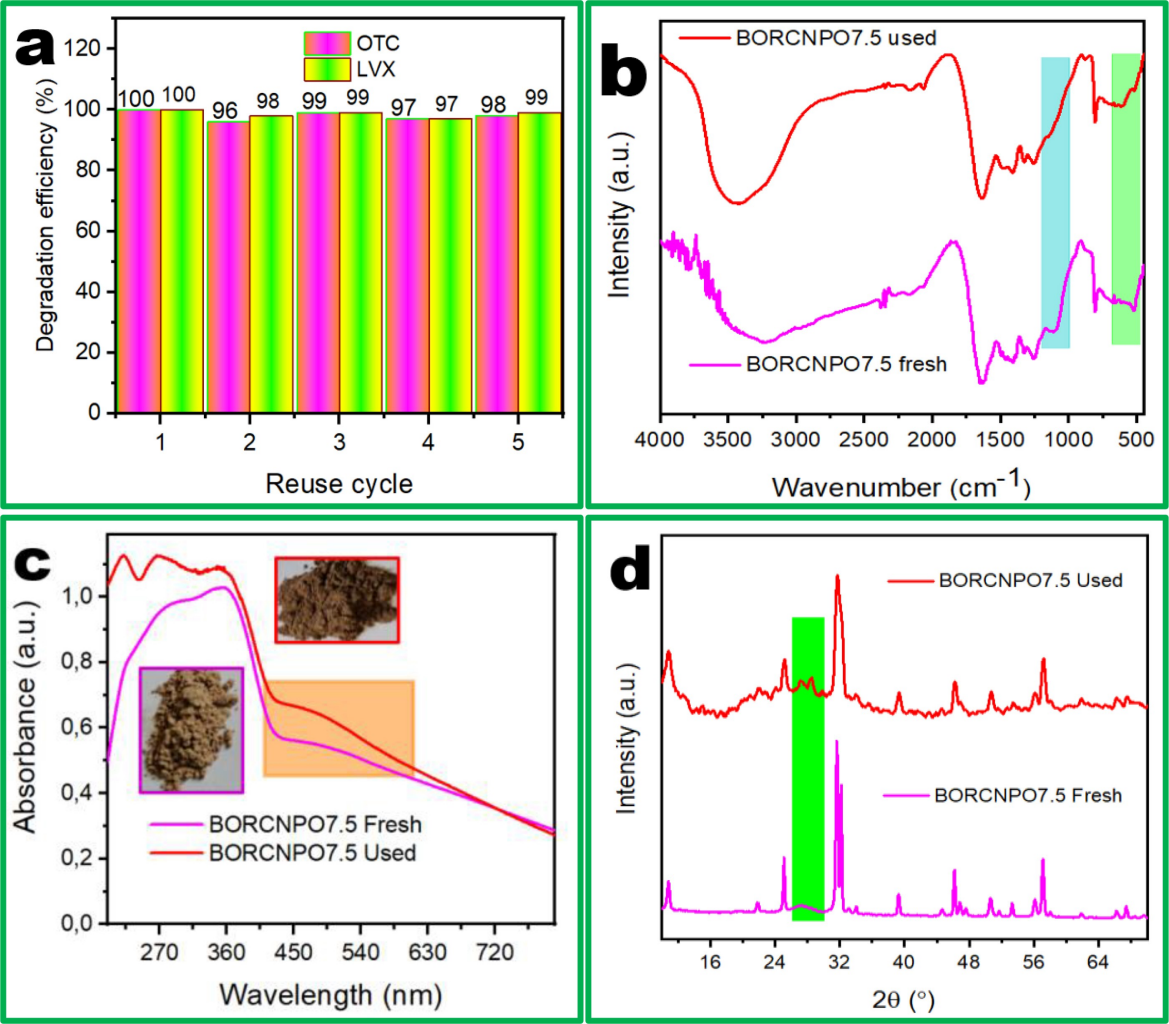
(a) Recycle experiments of BORCNPO7.5 at pH 6.32. (b) FTIR, (c) UV‐Vis, (c insert) pictures, and (d) XRD spectra comparisons between fresh and used catalyst.

The stability of the catalyst was assessed through cycling experiments. The photocatalyst was retrieved from the solution with centrifugation and dried without any washing for subsequent experiments. Figure 5a shows that the degradation efficiency of OTC decreased slowly after the first, second and third recycle tests with overall efficiency loss less than 5 % and increased to almost similar efficiency in the fourth and fifth cycle. In essence, the catalyst reactivity did not change resulting in a highly stable catalyst. The changes in bonding and functional groups of the BORCNPO7.5 used sample was studied with FTIR and compared with FTIR of fresh catalyst. Figure 5b demonstrated no change in FTIR spectra of fresh and used catalysts except the disappearance of the peak at 11123 cm^−1^. This peak disappearance could deduce that catalyst and pollutants interaction occurred through the C−N induced hydrogen bonds for efficient degradation.

Since XPS demonstrated the presence of plasmonic Bi metal, the reused sample was compared physically with the fresh one and there were observable colour changes. In Figure 5c (insert), the light brown colour of the fresh catalyst showed colour changes to dark brown after five cycling experiments. The observed changes in colour were attributed to the presence and increase in concentration of plasmonic Bi metal. UV‐Vis DRS comparison of fresh BORCNPO7.5 and its used counterpart supported the plasmonic effect suggestion. Plasmonic Bi metal absorbs light from 380 nm to the NIR region of the solar spectrum.[^49^, ^50^] As the catalyst was re‐used, metallic Bi concentration increased due to low redox cycles of Bi^3+^ to Bi^0^ and improved the ability of BORCNPO7.5 to absorb more visible light as depicted in Figure 5c. The improvement of the plasmonic effect assisted the enhanced generation of ROSs involved in the degradation of LVX and OTC which resulted in the increasing degradation efficiency after three cycles. This could imply that during the fourth cycle, the concentration and contribution of Bi SPR was high enough to musk out any catalyst deactivation mechanisms on the catalyst surface. Moreover, further increase in the efficiency of LVX and OTC degradation during the fourth cycle demonstrated that the SPR had not reached its optimum after five cycles and could still further improve the degradation rate and efficiency of OTC and LVX.

Figure 5d shows the XRD of the fresh and recycled catalysts. The crystallite phase of the photocatalyst was unchanged after recycling tests which proved its stability with new peaks at 2θ of 27° and 28° observed. In the fresh catalyst, this region showed a hump like that of an amorphous material which further resolves into the distinctive peaks after five cycles. These peaks matched well with 003 planes of metallic Bi proving that it is polycrystalline.[^28^, ^50^] The broadness of the XRD peaks increased after reuse cycles, but their intensity decreased. Peak broadness could further demonstrate that Bi metal doping concentration increased in BOR after cycling experiments. This is because all the peaks in the composite were due to BOR and since BOR was proved to be a RP, it improved reduction potential of BORCNPO7.5. Therefore, the results demonstrated that the stability and activity of BORCNPO7.5 was enhanced by plasmonic metallic Bi formation which increased rate of LVX and OTC degradation with time.

### Photodegradation Mechanism of BORCNPO7.5

**Figure 6:**
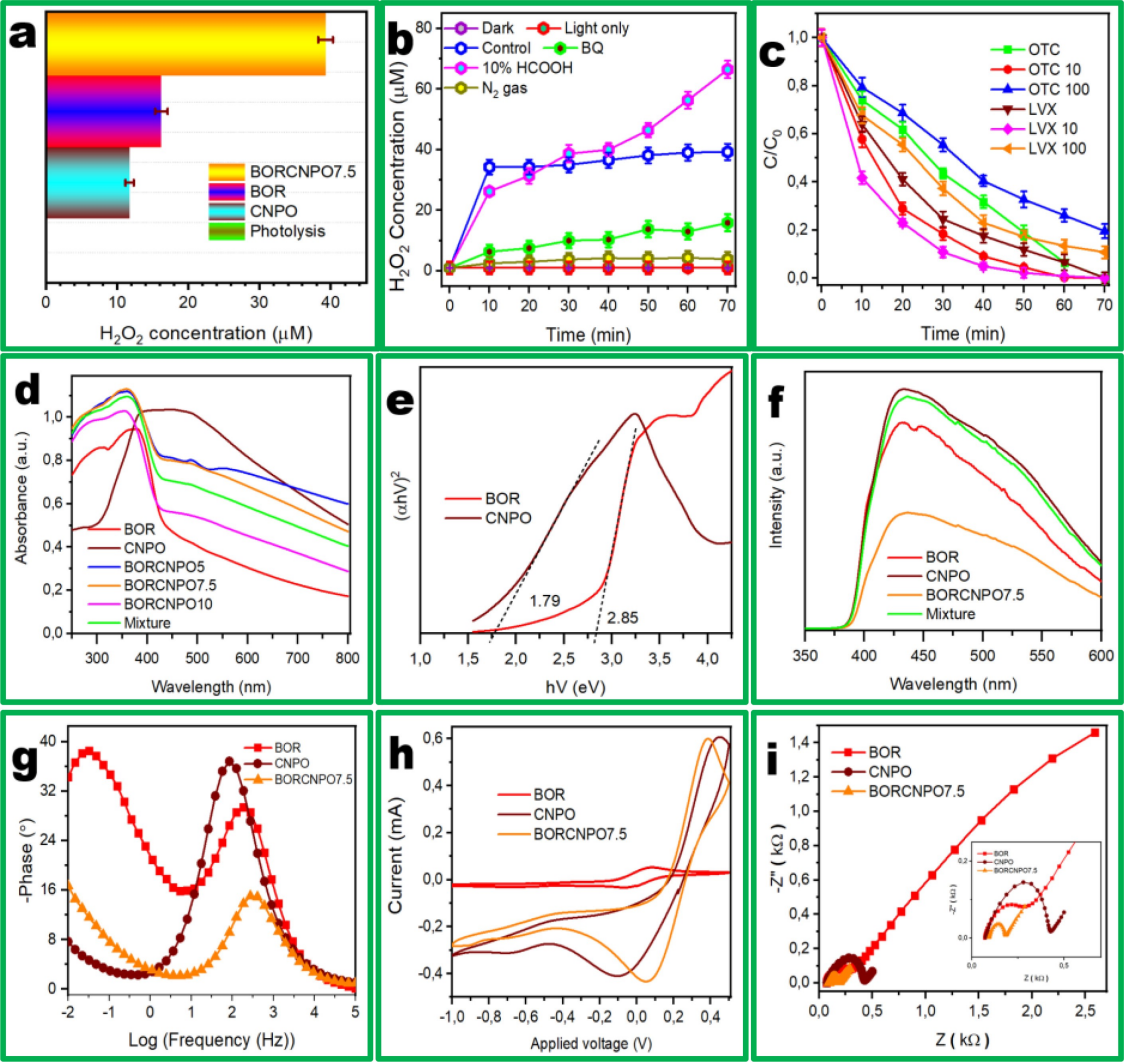
H_2_O_2_ production (a) activity of different catalysts, (b) changes in conditions, and (c) influence of H_2_O_2_ amount on LVX and OTC degradation with BORCNPO7.5. (d) UV‐vis DRS, (e) Tauc plot, (f) PL, (g) Phase, (h) EIS, and (i) CV of composites.

Investigation of the degradation mechanism pathway of the S‐scheme heterojunction was examined through analysis of in‐situ H_2_O_2_ production from the sampled aliquots at different time intervals using potassium titanium (IV) oxalate spectrophotometric method. Figure 6a demonstrated that H_2_O_2_ production was not observed during photolysis without catalyst, while CNPO, BOR, and BORCNPO7.5 generated H_2_O_2_ in 70 min. BORCNPO (39.31 μM) generated 3.4 and 2.4 times the H_2_O_2_ concentration than CNPO (11.69 μM) and BOR (16.13 μM), respectively. The corresponding zero‐order formation rate constants (K_f_) in Figure S4a obtained from$\left[H_{2}O_{2}\right]=\frac{K_{f}}{K_{d}}\left(1-e^{K_{d}t}\right)$ ^[51]^ supported that the H_2_O_2_ production rate occurred in the order BORCNPO7.5>BOR>CNPO. The S‐scheme heterojunction improved the generation of H_2_O_2_.

The photocatalytic production of H_2_O_2_ concurrently involves its decomposition by photogenerated holes and electrons on the catalyst. More investigation on the decomposition of H_2_O_2_ were conducted and Figure S4b shows that the BORCNPO7.5 catalyst resulted in a high decomposition of H_2_O_2_. When comparing the pseudo first‐order decomposition rates (K_d_) of the catalysts in Figure S4c, the S‐scheme BORCNPO7.5 gave high K_d_ of 0.01548 min^−1^ that was 1.43 times higher than that of BOR (0.01084 min^−1^) and 2.98 times more than that of CNPO (0.00519 min^−1^). Comparison of both the K_f_ and K_d_ in Figure S4d showed that BORCNPO7.5 had the highest K_f_ and K_d_ which showed that the formed H_2_O_2_ was quickly decomposed into ROSs for increased pollutant mineralization rate. This observation was supported by the results that were reported by Zhao et al.^[52]^ High K_f_ versus low K_d_ demonstrates high formation of H_2_O_2_ as in CNPO while high K_f_ and high K_d_ demonstrated efficient utilization of in‐situ H_2_O_2_ to form ROSs for enhanced degradation of the pollutants as in BORCNPO7.5. Therefore, the fabrication of the S‐scheme heterojunction increased the formation and utilization of H_2_O_2_ in‐situ for efficient degradation.

Different conditions were investigated to understand the mechanism behind the in‐situ H_2_O_2_ production by BORCNPO7.5 with light only without catalyst, catalyst under dark, BQ as electron scavenger, 10 % HCOOH as holes scavenger, and under N_2_ gas environment. As seen from Figure 6b, there is a steady increase in formation of H_2_O_2_ which became constant when the formation and decomposition equilibrium of H_2_O_2_ is reached in the control experiment. Similar results were observed by Ma et al.^[17]^ When only light and the catalyst in the dark experiments were conducted, H_2_O_2_ could not be produced to demonstrate the photocatalysis requirement of H_2_O_2_ generation. Moreover, the used source of light could not decompose 2 mM H_2_O_2_ as shown in Figure S4c. To understand the underlying mechanism for H_2_O_2_ production, scavenger tests were conducted to index it to either electron reaction or water oxidation reaction (2H_2_O+2 h^+^→H_2_O_2_+2H^+^, E^0^=+1.76 V).^[53]^ The results for BORCNPO7.5 catalyst in 10 % methanol solution (Figure 6b) demonstrated increase in H_2_O_2_ production. The increased production rate when holes were scavenged with HCOOH portrayed the major contribution of the direct and indirect electron reactions (Equation (1)).

When BQ was used as electrons scavenger, H_2_O_2_ production rate dropped significantly to show the importance of electron reaction in this system. But little H_2_O_2_ production occurred which demonstrated the existence of another pathway of H_2_O_2_ formation. Under inert N_2_ gas, there is significant decrease in H_2_O_2_ production which supported the proposition for the electron transfer mechanism (Equation (1)). This further demonstrated the requirement of oxygen and supported the electron reduction process as the main mechanism of H_2_O_2_ production. Therefore, in‐situ H_2_O_2_ occurred mostly through the electron reduction reaction. However, water oxidation did occur which was believed to be catalysed by h^+^ at the rock water interface and defects that form oxygen radical (O⋅).^[54]^ The O⋅ is responsible for the direct water oxidation to H_2_O_2_ (H_2_O+O⋅→H_2_O_2_). Therefore, CNPO morphology also influenced the formation of in‐situ H_2_O_2_ in BORCNPO7.5 resulting in enhanced overall production of H_2_O_2_ than the BORCNPO7.5 (Figure S4d).

The consumption of H_2_O_2_ by the BORCNPO7.5 catalyst was investigated through monitoring the degradation of LVX and OTC with time when external H_2_O_2_ is added to further support the hypothesis that H_2_O_2_ was decomposed into ROSs in BORCNPO7.5. The different concentrations of H_2_O_2_ were externally added to investigate their effect on the degradation of LVX and OTC. Figure 6c showed that additional 10 μM H_2_O_2_ increased the degradation rate of OTC and LVX. The increased degradation rate can be indexed to the presence of H_2_O_2_ as the major source of and which was converted to ⋅OH (H_2_O_2_+e^−^→⋅OH+OH^−^).^[55]^ Except the involvement of electrons in H_2_O_2_ decomposition, Fenton like H_2_O_2_ with low redox Bi can be another mechanism. The increased concentration of Bi^0^ can be envisaged to occur through electron reduction of Bi^3+^. This would increase surface plasmon resonance and supply enough Bi^0^ for H_2_O_2_ decomposition (Bi^0^+H_2_O_2_→Bi^3+^+2⋅OH). Moreover, the regeneration reaction occurs to complete the Bi^0^/Bi^3+^ redox cycle.^[56]^

Lastly, oxygen vacancies have been reported to act as Fenton catalytic centres for H_2_O_2_ decomposition^[56]^ and their presence in BORCNPO7.5 was believed to have contributed to its dissociation to ROSs. The final degradation rate of LVX and OTC in the presence of additional H_2_O_2_ was almost completed in 50 min. This affirmed that the addition of H_2_O_2_ increased the rate of ⋅OH production which enhanced pollutants degradation. Further increase of H_2_O_2_ to 100 μM decreased the degradation rate and the final removal efficiency of OTC and LVX. The decreased reaction rate when excess H_2_O_2_ was added can be described in terms of the H_2_O_2_ scavenging activity of ROSs which eventually retards their rate of degradation (H_2_O_2_+⋅OH→H_2_O+HO_2_⋅).^[57]^ This would occur as H_2_O_2_ would not be completely decomposed into ROSs and the excess one will then scavenge the present ROSs. Lastly, H_2_O_2_ would also scavenge the photoexciton pairs that are responsible for generation of ROSs. Therefore, in excess H_2_O_2_, formation of ROSs decreases which lowered degradation rate. Hence, BORCNPO7.5 produced H_2_O_2_ in‐situ which was decomposed into ⋅OH and its contribution with other ROSs resulted in the observed degradation of OTC and LVX.

The catalysts optical and electrochemical properties were investigated to comprehend the observed degradation and H_2_O_2_ generation capability. The UV‐Vis DRS of samples is shown in Figure 6d, CNPO had strong visible light absorption while BOR had a strong UV absorption and had an absorption edge in the visible region around 490 nm. The mixture of CNPO and BOR resulted in the poorest light absorption intensity in the visible light. Incorporation of CNPO and BOR increased the light absorption capability of BOR with observable red shift of its absorption edge to lower energy region. To affirm the importance of catalyst composition optimization, BORCNPO7.5 demonstrated two peak humps at 488 and 560 nm indexed to presence of plasmonic Bi.^[28]^ These peaks were not observed in BOR and demonstrated the absenteeism of surface plasmon resonance effect as supported by XPS analysis. The results affirmed plasmonic Bi presence towards expansion of visible light response in S‐scheme BORCNPO7.5. The bandgap energy (E_g_) of BOR and CNPO were determined with the relationship presented in Equation 11.

(11)

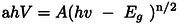




where A, α, and hV are absorption coefficient, and photon energy. In Figure 6e, the E_g_ of BOR and CNPO were 2.85 and 1.79 eV, respectively. PL results in Figure 6f demonstrated that the fabricated BORCNPO7.5 heterojunction has less carrier recombination than BOR and CNPO. The mixture of BOR and CNPO had a slightly lower peak, but it was as pronounced as the CNPO indicating that a physical mixture hardly decreased the probability of electrons and holes pairs recombination due to lack of interfacial contact. BOR had a lower PL emission peak than CNPO demonstrating efficient charge separation that was attributed to presence of defect OVs that trapped excited electrons. The formation of heterostructure proved valuable towards effective improvement of lifetime of exciton pairs.

Electrochemical techniques were deployed to further comprehend the internal charge transfer properties of the catalysts. Bode phase angle graph demonstrated that CNPO had lower phase angle than BORCNPO7.5 which demonstrated that BORCNPO7.5 possessed higher capacitance aligned to its improved ability to store charge as shown by Figure 6g. The increased charge storage capability directly correlated to extended lifetime of electrons though it has been reported that it is not easy to approximate.^[58]^ In Figure 6h, CV of composites was compared and BOR had the redox peaks indexed to low redox capability than CNPO with high redox peak currents with oxidation peak around 0.4 and reduction peak maximum at −0.1 eV. In the S‐scheme BORCNPO7.5 composite, the oxidation peak current of CNPO was maintained while its reduction peak maximum increased. This could be indexed to the formation of S‐scheme heterojunction as type II charge transfer results in reduction of redox capability of pristine semiconductors.^[7]^ Moreover, peak shifts occurred which demonstrated that BOR improved the oxidation and reduction ability of CNPO due to formation of the heterojunction. Figure 6i demonstrated EIS spectra results which was used to interrogate the electron transfer resistance behaviour of composites. Figure 6i insert showed that the arc radius of CNPO was higher than BOR and fabrication of BORCNPO7.5 resulted in lowest arc radius at low frequencies. The lower arc radius of BORCNPO7.5 implied low charge transfer resistance resulting from the construction of the S‐scheme heterostructure. This could be indexed to improved movement of charge due to efficient separation which enhanced the redox capability of the composite.

**Figure 7:**
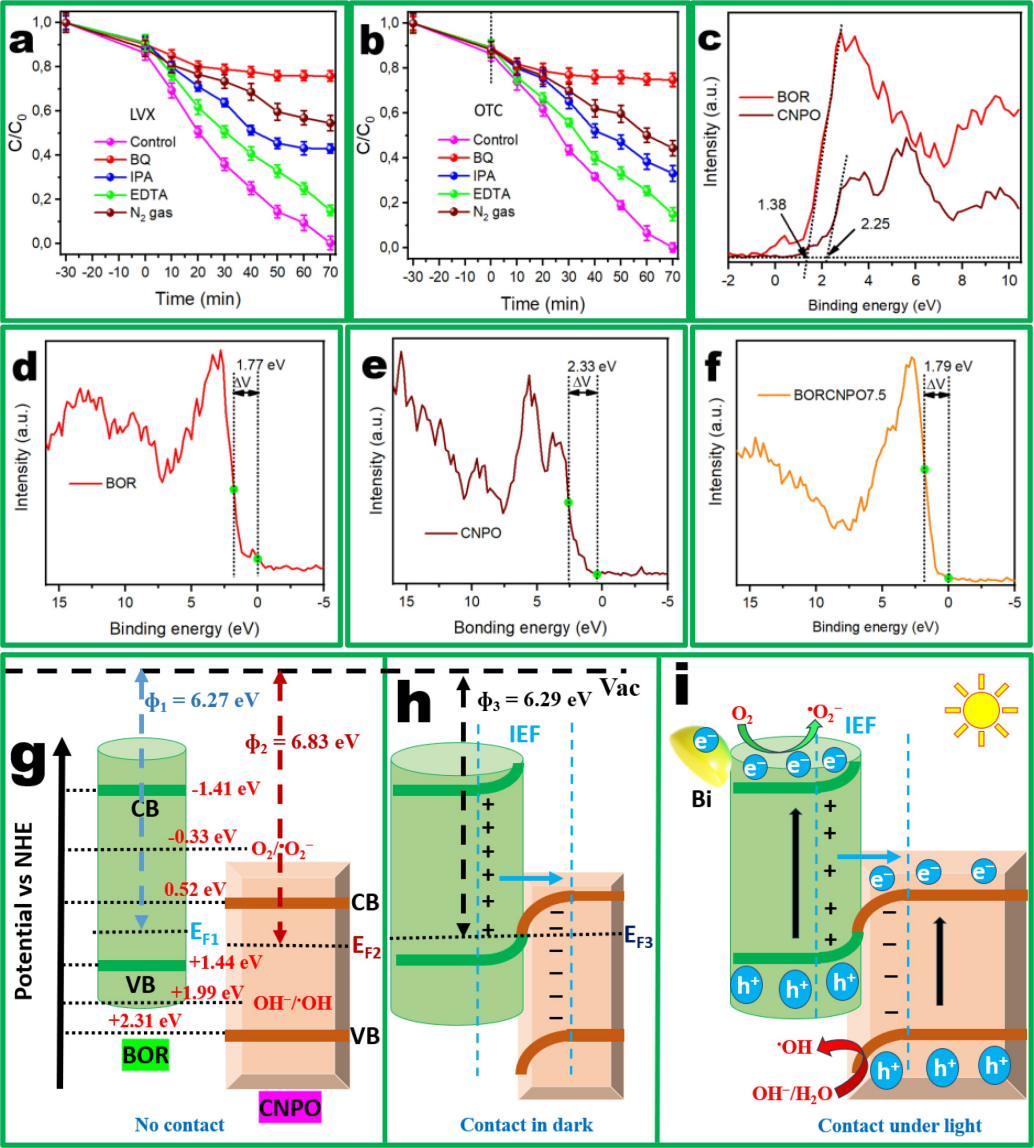
Radical trapping experiments of BORCNPO7.5 during degradation of (a) OTC, and (b) LVX. (c) XPS‐VB of BOR and CNPO, XPS‐VB work functions of (d) BOR, (e) CNPO, and (f) BORCNPO7.5. (g) Band structure of BOR and CNPO, (h) interaction of BOR and CNPO under dark, and (i) Proposed S‐scheme charge transfer under light irradiation for BORCNPO7.5.

Radicals scavenging tests were conducted to further elucidate the presence and involvement of ROSs, especially superoxide (⋅O_2_^−^), hydroxyl radicals (⋅OH) and photoinduced holes (h^+^) during BORCNPO7.5 heterojunction deployment. During LVX and OTC degradation, IPA, EDTA, and BQ were used as trapping agents of ⋅OH, h^+^ and ⋅O_2_^−^, respectively. Figure 7a showed that LVX degradation was affected by all radicals with the major reduction in degradation rate observed when BQ was used while EDTA resulted in the lowest rate reduction. Similarly, OTC degradation occurred through the same sequence with almost equal magnitude as depicted in Figure 7b. This demonstrated the uniformity of generated ROSs which were independent of the pollutant. N_2_ gas was bubbled to substitute O_2_ and provide an inert environment for the photocatalytic degradation process during LVX and OTC degradation. The efficiency decreased but the observed degradation efficiency could be attributed to the presence and involvement of both ⋅OH and h^+^ as ⋅O_2_^−^ would form in the presence of O_2_. The generated ROSs supported the formation of the S‐scheme mechanism with distinctive OP (CNPO) and RP (BOR) as determined. In essence, ⋅O_2_^−^ played the major role as the LVX and OTC removal efficiency conspicuously decreased after the addition of BQ. IPA resulted in considerable decrease in efficiency and demonstrated that ⋅OH was involved in the degradation of pollutants. Lastly, parsimonious involvement of h^+^ was devoted to small reduction of LVX and OTC degradation rate in the presence of EDTA.

The energy band structure of BOR and CNPO was investigated as an important tool towards understanding potential generation of ROSs. To determine the band structure of pristine BOR and CNPO, their respective valence band energy (E_VB_) and conduction band energy (E_CB_) should be determined. Figure 7c showed the XPS valence band (XPS‐VB) spectra of BOR and CNPO with respective values of 1.38 and 2.25 eV. The E_VB_ value is normally determined from relationship with E_XPS‐VB_ in Equation 12., ^[59]^

(12)

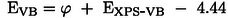



(13)

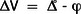




where φ (4.5) is the electron work function of the XPS analyzer. The E_VB_ of BOR and CNPO were 1.44 and 2.31 eV and their E_CB_ calculated from (E_CB_=E_VB_ − E_g_)^[60]^ were −1.41 and 0.52 eV for E_g_ values of 2.85 and 1.79 eV, respectively. The catalysts work function (φ) can be calculated from XPS‐VB curve contact potential difference (ΔV), which denotes the difference of two variation points (IP) using Equation (13)[^61^, ^62^] to estimate the fermi level energy (E_F_). From Figure 7d–f, φ of BOR, CNPO, and BORCNPO7.5 were estimated to be 6.27, 6.83, and 6.29 eV, respectively. From the respective positions of E_F1_ and E_F2_, BOR and CNPO are *p‐*type and *n‐*type semiconductors as their E_F_ are close to their VB and CB, respectively.^[17]^ When BOR and CNPO are in contact, due to the high fermi energy level of BOR (E_F1_) than that of CNPO (E_F2_), electrons will transfer from BOR to CNPO. The band energies of CNPO bends downward while those of BOR bended upwards resulting in electron accumulation layer at the interface of CNPO and an electron depletion layer at the interface of BOR pioneered by the electron movement.

Consequently, an internal electric field (IEF) pointing from BOR to CNPO is formed such that under light illumination, the electron depletion on BOR towards the interface region influences upwards bend bending while the electron accumulation on CNPO influences downward bend bending. Due to this bend bending, the electrons flow along the gradient is induced and the IEF induces photogenerated electrons transfer from the CB of CNPO to the VB of BiOBr to recombine with photogenerated holes. This achieves efficient charge separation and ensures that electrons in the CB of BOR with high reduction capability are involved in pioneering formation of ROSs through reduction reactions while holes in the VB of CNPO with strong oxidation potential initiated oxidation reactions for enhanced ROSs formation.^[63]^ Therefore, oxidation and reduction reactions occurred at distinctive oxidative and reductive semiconductor sites with suitable stability to both oxidation and reduction reactions, respectively.

From Figure 7g, the band structures of CNPO and BOR were staggered which supported formation of S‐scheme heterojunction with CNPO being the oxidative photocatalyst (OP) while BOR was the reductive photocatalyst (RP). Investigations that report BOR based catalysts have shown that it can be either oxidative or reductive.[^31^, ^64^, ^65^] The synthesis method and precursors used in this method are believed to form a very reductive BOR. However, g‐C_3_N_4_ is normally reductive in nature but just recently, studies on P doped g‐C_3_N_4_ has gained attention with few articles reporting its oxidative nature.[^31^, ^66^] In this work, the oxidative CNPO is believed to be influenced by the quantity of P dopant.

XPS analysis was used to support the proposed S‐scheme heterojunction. The BE of C, N, and P of CNPO increased in the dark while BOR Bi and Br BE decreased in BORCNPO7.5 as illustrated in Figure 3b–f. The shifts to lower BE established higher electron density when CNPO is in contact while BOR BE increased to confirm its low electron density due to loss of electrons during contact when the heterojunction is formed. Therefore, electrons movement during contact in the dark was established to occur from BOR to CNPO such that BOR has electron depletion and become positively charged at the interface with CNPO which has a negative charge. The charges result in built‐in internal electric field (IEF) that is from BOR to CNPO and bend bending occurs as shown by Figure 7h.[^31^, ^34^] Under irradiation, BOR and CNPO absorb light of appropriate wavelength and results in electrons and holes pairs generation Figure 7i. The separation of these photogenerated charges is achieved through interfacial recombination of electrons in the CB of CNPO and holes in the VB of BOR. To further investigate the interfacial recombination and probable S‐scheme heterojunction formation, XPS was used to postulate charge transfer behaviour.

**Figure 8:**
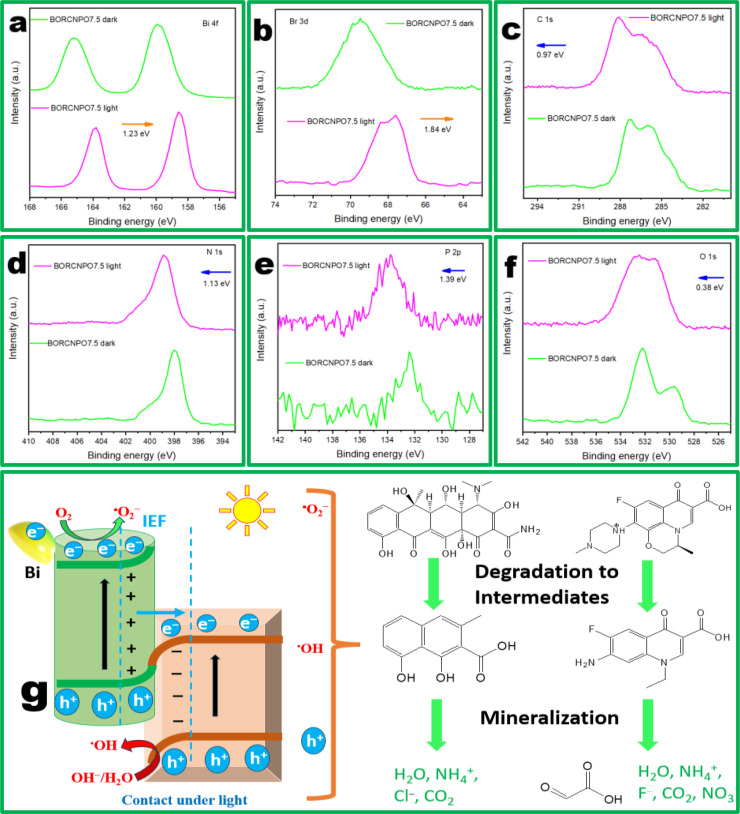
XPS high resolution of BORCNPO7.5 under dark and light for (a) Bi 4 f, (b) Br 3d, (c) C 1s, (d) N 1s, (e) P 2p, and (f) O 1s. (g) Degradation mechanism of OTC and LVX by BORCNPO7.5.

The comparison of XPS BE of BORCNPO7.5 in dark and under light gave insights into the charge transfer direction under light as their survey scan showed no changes in elemental composition in Figure S5 with presence of Bi 4 f, Br 3d, C1s, N 1s, P 2p, and O 1s. From Figure 8a and b, the BE of Bi 4 f and Br 3d decreased with respective magnitude of 1.36 and 1.84 eV, respectively. The decrease in BE demonstrated increase in electron density as electrons were proposed to accumulate on the VB of BOR under light irradiation. Alternatively, BE of C 1s, N 1s, and P 2p in Figure 8c and e increased under light irradiation with corresponding magnitude of 0.97, 1.13, and 1.39 eV respectively. This BE positive change could be indexed to low electron density envisaged to occur as CB electrons on CNPO are transferred to VB of BOR. However, the BE magnitude of O 1s increased with 0.38 eV (Figure 8f) and demonstrated low electron density as the bridging C−O−Br transports electrons. Therefore, XPS provided a plausible explanation of the charge transfer pathway as S‐scheme. Interestingly, the O 1s spectra demonstrated an increase in BE from BOR and CNPO to BORCNPO7.5 under dark. This further supported the formation of the C−O−Br bridging bond and its involvement towards the formation of the proposed S‐scheme heterojunction. The bridging bond accepts electrons and transfer them and acts as an efficient electron pathway for separation of photoexciton charges. This cemented the PL and electrochemical charge transfer efficiency analysis.

Assuming degradation of LVX and OTC by BORCNPO7.5 followed a traditional type II mechanism, the generation of ⋅OH and ⋅O_2_^−^ would not be possible. This is because in a traditional type‐II charge transfer as depicted in Figure S6, electrons will be envisaged to move from the E_CB_ BOR and accumulate on the E_CB_ of CNPO while holes will move from E_VB_ of CNPO and accumulate on E_VB_ of BOR when in contact under light irradiation.^[67]^ Based on the E_CB_ of CNPO and E_VB_ of BOR, electrons on E_CB_ of CNPO have more positive potential than the potential required to generate ⋅O_2_^−^ (E vs. NHE of O_2_/⋅O_2_^−^ is −0.33 eV) and the E_VB_ of BOR with a potential of +1.44 eV would have inadequate energy for OH^−^/H_2_O oxidation into ⋅OH (E vs. NHE of OH^−^/⋅OH is +1.99 eV or H_2_O/⋅OH is +2.3 eV).^[68]^ Additionally, based on XPS charge transfer results, the electrons movement in this direction would be impossible as it would go against the IEF direction obtained when XPS measurements were done under dark conditions.^[69]^ Of note, the type II heterojunction formation contradicted with the trapping experiments results that demonstrated existence and involvement of ⋅O_2_^−^ and ⋅OH. This evidently ruled out the type II heterojunction formation leaving the S‐scheme heterojunction as the plausible alternative.

In the proposed S‐scheme heterojunction, both CNPO and BOR absorbs energy equivalent to their bandgaps and resulted in formation of photoexciton pairs. Electrons get excited and accumulate on their lower CB levels leaving holes on the VB. Influenced by the IEF and band bending that occurs, electrons in the CB of CNPO recombines with holes on the VB of BOR and resulted in efficient charge separation. Moreover, more charge separation efficiency can be achieved as the OV of BOR provided an impurity level and modulated excitation of electrons from its VB to the impurity level.^[31]^ Since BORCNPO7.5 had Bi surface plasmonic effect with concentration that increased with photodegradation time, more light would be absorbed and results in more electrons being generated by the RP (BOR) (Figure 8g). Evidently, the high influx of generated electrons on CB of BOR, OV of BOR and due to SPR would collectively results in formation of more ⋅O_2_^−^. This would result in ⋅O_2_^−^ being the main radical involved in LVX and OTC degradation.

From the band structures of CNPO and BOR, they can both results in formation of H_2_O_2_ E(O_2_/H_2_O_2_, 0 : 68 eV/NHE). In addition, h^+^ in the VB of CNPO would react with OH^−^/H_2_O to form ⋅OH or H_2_O_2_ intermediate. Another H_2_O_2_ formation mechanism in this system could be through a two electron process (Equation (1)). Interestingly, the decomposition of H_2_O_2_ improves the mineralization capability of BORCNPO7.5 through production of more ⋅OH that aided mineralization of LVX and OTC. The presence and increase of Bi^0^ could assist with enhanced conversion of H_2_O_2_ into ⋅OH though low redox cycles of Bi from Bi^0^ →Bi^3+^ which then is reduced to Bi^0^ (Equations (14) and (15)). Lastly, h^+^ in the VB of both BOR and CNPO would also be involved in the degradation of pollutants with ⋅OH and ⋅O_2_^−^ to form intermediates that are further mineralized into aliphatic compounds, H_2_O, and CO_2_ (Equation 16.
(14)

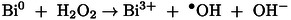



(15)

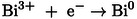



(16)

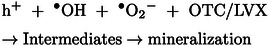




### Intermediates Analysis and Degradation Pathway of OTC and LVX

As established from previous experiments, degradation of OTC and LVX occurred due to generated ROSs (⋅O_2_^−^, ⋅OH, H_2_O_2_, and h^+^) which resulted in their attack on several bonds of the parent molecules. Moreover, 3D FEEM images demonstrated existence of fluorescing intermediates after 70 min of irradiation time, most of which were identified by QTOF‐HPLC‐MS in positive mode from the reaction samples at different time intervals. The spectra of the intermediate products (IP) analysis for OTC are presented in Figure S7a–d. Depending on the m/z values observed, chemical structures of intermediate products (IP) were assumed and used for the proposed OTC degradation pathways (PW). ROSs are electrophilic materials and their attack on organic molecules favourably occurs on more frontier electron rich sites according to the Frontier Orbital Density theory.^[70]^ Similar behaviour is expected to occur during LVX degradation which would results in different pathways. The pathways were speculated with detected IP (black font colour) and undetected IP (red font colour) where the latter was obtained from literature.[^71^, ^72^]

**Figure 9:**
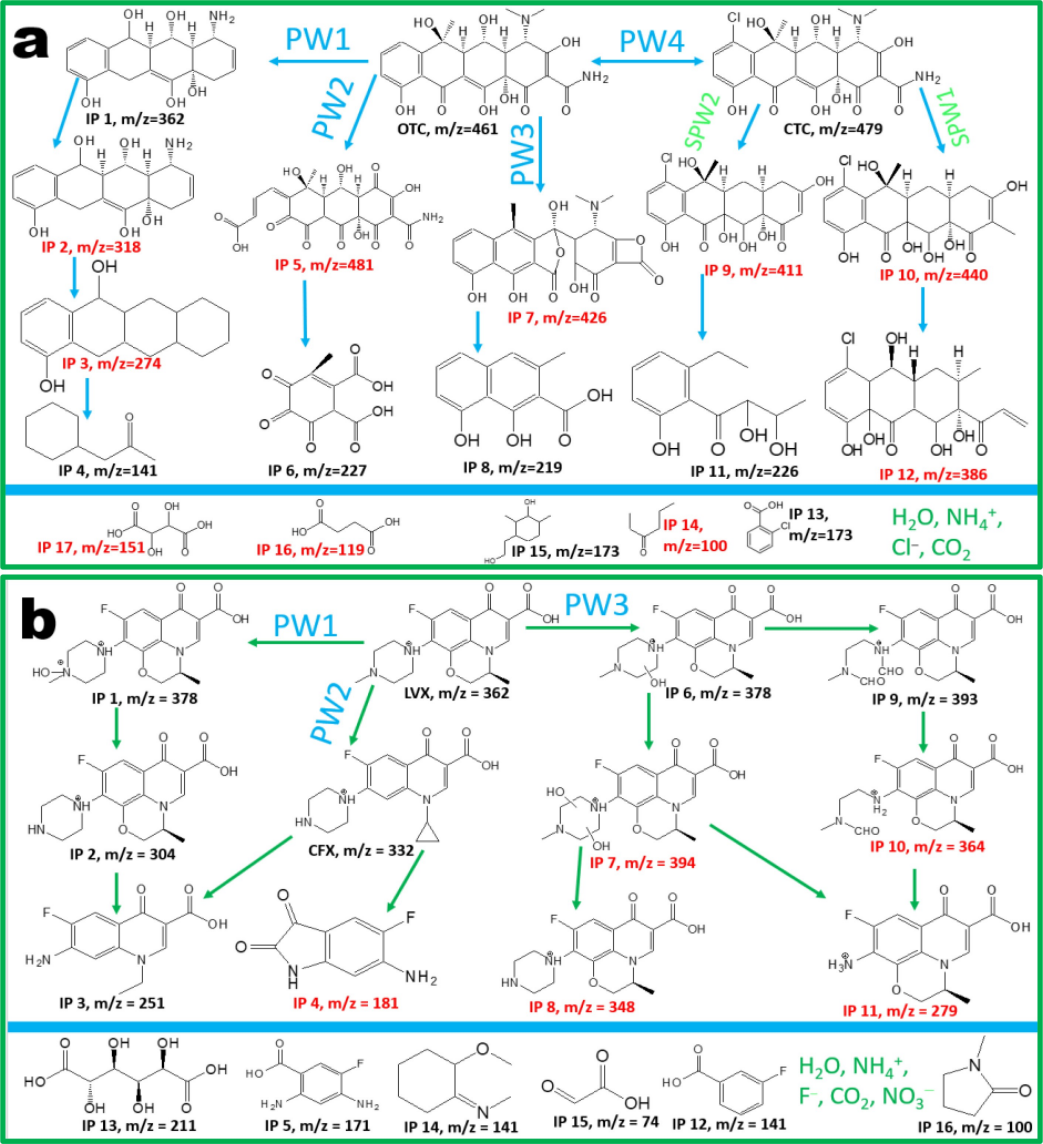
Degradation pathway and intermediate products during degradation of (a) OTC and (b) LVX with BORCNPO7.5.

Figure 9a shows Pathway 1 (PW1) which is envisaged to occur first through the loss of hydroxyl, carbonyl and N‐methyl groups accompanied by deamidation reaction into IP 1 (m/z=362). IP 1 is degraded into IP 2 (m/z=318) through methyl, hydroxyl, and carbonyl functional groups losses while IP 2 was transformed into IP 3 (m/z=274) through oxidative dihydroxylation and deamination as reported in literature.^[40]^ Further oxidation of IP 3 resulted in benzyl and non‐benzyl ring opening reactions which lead to formation of low molecular weight detected IP 4 (m/z=141). PW 2 proceeded through the ring opening reaction on the phenol hydroxyl to form IP 5 (m/z=481) that was further oxidized into IP 6 (m/z=227).^[72]^ PW 3 was envisaged to occur through hydrolysis and deamination of OTC to IP 7 (m/z=426) which was further oxidized through the cleavage of the C−C bridging bond connecting two heterocyclic compounds.^[73]^ The oxolane heterocyclic compound would incur ring opening reactions on the oxolane functional group with hydroxylation to IP 8 (m/z=219).

Interestingly, the OTC was chlorinated in solution with the observed IP (m/z=279) identified as chlortetracycline (CTC) resulting in PW 4. Based on the detected IP and literature,^[39]^ two CTC degradation PW were assumed to occur as SPW 1 and SPW 2. The SPW 1 involved demethylation, deamination, and hydroxylation reactions to form IP 10 (m/z=440) that is further oxidized through ring opening reaction to form IP 12 (m/z=386). Alternatively, SPW 2 involved deamidation and demethylation into IP 9 (m/z=411) which was further degraded through a couple of ring opening, dechlorination, decarboxylation, dehydrogenation to form IP 10 (m/z=226).^[39]^ The low molecular weight IP attained through the degradation pathways of OTC which included CTC formation and degradation resulted in formation of IP with overlapping m/z compounds (i. e. IP 13 and IP 15 with m/z=173) resulting in the high intensities of the by‐products. These high intensities were assumed to musk out other IP like IP 14 (m/z=100), IP 16 (m/z=119), and IP 17 (m/z=151). Interestingly, the hump observed at 475 nm during OTC degradation. could be the result of CTC presence. Further oxidation of the low molecular weight compounds resulted in their mineralization to organic acids and nitrogen or chlorine containing by‐products including water and carbon dioxide as reported in literature.[^39^, ^72^]

The LVX QTOF‐HPLC‐MS spectra presented in Figure S8a–d was used with literature to propose the most plausible degradation pathway of LVX with BORCNPO7.5.^[45]^ The photodegradation of LVX followed three pathways that were deduced based on the m/z values of the observed intermediate products as presented in Figure 9b. PW1 involves the hydroxylation of LVX on the N atom to IP 1 (m/z=378) which is very unstable and would be dehydroxylated and demethylated into IP 2 (m/z=304).^[45]^ Further attack and removal of the piperazinyl ring and the attack on the oxygen atom of the heterocyclic ring resulted in IP 3 (m/z=251) which was also believed to occur through PW2. In PW2, the initiation of LVX degradation occurred through the demethylation of the piperazinyl ring and ring opening of the oxolane functional group on the heterocyclic ring which formed a ciprofloxacin (CIP) with m/z=332. The CIP degradation pathways reported in literature has been reported to form IP 3 with m/z=251 through piperazinyl ring removal and the cyclopropane ring opening. Another product could be formed by the attack on CIP that started with decarboxylation, removal of piperazinyl ring, and removal of the cyclopropane ring. Ring opening and carboxylation of the side that has the cyclopropane and rearrangement resulted in formation of IP 4 (m/z=181) which would be decarboxylated after ring opening of the five‐membered heterocyclic ring to give IP 5 (m/z=171).^[45]^ Deamination of IP 5 can result in formation of 4‐Flurobenzoic acid (IP 12, m/z=141).

PW3 occurred through hydroxylation of the piperazinyl ring of LVX and gave IP 6 (m/z=378) which was broken down through two possible ways. In the first instance, IP 6 is hydroxylated on the piperazinyl ring to give IP 7 (m/z=394) which would be dehydroxylated and demethylated into IP 8 (m/z=348).^[74]^ The second way can be through the piperazinyl ring opening and carboxylation into IP 9 (m/z=393). The demethylation of one side of the ring resulted in the formation of IP 10 (m/z=365) which when demethylated and deaminated formed IP 11 (m/z=279).^[75]^ From PW1, PW2 and PW3 during LVX degradation, smaller molecules that were observed (Figure S8c and d) demonstrated the mineralization of LVX occurred through formation of intermediate products that were further degraded. IP 13 (m/z=211), IP 14 (m/z=141), IP 15 (m/z=74), and IP 16 (m/z=100) were formed and further mineralized into water, carbon dioxide, anions (fluoride, nitrate), and cationic ammonium ion as reported.[^75^, ^76^] The degradation of LVX demonstrated the capability of the synthesized BORCNPO7.5 towards degradation of fluoroquinolone antibiotics with formation and degradation of CIP while it also demonstrated high capability towards degradation of tetracycline antibiotics with degradation of OTC which occurred with CTC intermediate pathway formation and destruction into low molecular weight and less hazardous intermediate products. Therefore, S‐scheme BORCNPO7.5 demonstrated ability towards degradation of different pollutants and can be recommended as a potential catalyst for environmental pollution remediation.

## Conclusions

In summary, an S‐scheme heterojunction between CNPO and BOR was triumphantly constructed through in‐situ hydrothermal method by mounting worm‐like 3D BOR on the surface of 3D cracked rock stone‐like CNPO with strong interaction through formation of C−O−Br bridging bond. The formation of bridging bond assisted as an electron pathway for efficient separation of photoexcited electrons and holes which enhances their lifetime as supported by PL and electrochemical characteristics. The efficient charge transfer is important for efficient charge separation during the construction of S‐scheme heterojunction which improved stability towards generation of ROSs for LVX and OTC mineralization. Moreover, SPR and OV on BOR improved generation of ⋅O_2_^−^ which acted as the major contributor followed by ⋅OH and h^+^ in the mentioned order as supported by trapping experiments. The S‐scheme formation was also supported by XPS analysis which was further used to find the band structure of the pristine composites for affirming a plausible mechanism. Moreover, in‐situ generation and consumption of H_2_O_2_ improved OTC and LVX and by‐products mineralization. FEEM and QTOF‐HPLC affirmed the degradation of both OTC and LVX with the rate being higher for the latter than the former. This work demonstrated a highly adaptive S‐scheme BOR and CNPO heterostructure that could be beneficial for degradation of organic pollutants and for green H_2_O_2_ production.

## Author Contributions

M. E. Malefane: Conceptualization, Methodology, Funding acquisition, Writing – original draft, Writing – review & editing. M. Managa: Resources, Writing – review & editing. T. T. I. Nkambule: Resources, Writing – review & editing. A. T. Kuvarega: Supervision, Writing – review & editing, Validation, Funding acquisition. The manuscript was approved by all authors.

## Conflict of Interests

No competing interests to declare.

1

## Supporting information

As a service to our authors and readers, this journal provides supporting information supplied by the authors. Such materials are peer reviewed and may be re‐organized for online delivery, but are not copy‐edited or typeset. Technical support issues arising from supporting information (other than missing files) should be addressed to the authors.

Supporting Information

## Data Availability

The data that support the findings of this study are available from the corresponding author upon reasonable request.
