# Supplementary material for: s‐scheme3D/3D Bi0/BiOBr/P Doped g‐C3 N4 with Oxygen Vacancies (Ov) for Photodegradation of Pharmaceuticals: In‐situ H2O2 Production and Plasmon Induced Stability
Source: ChemSusChem. 2024 Oct 18;18(2):e202401471. doi: 10.1002/cssc.202401471 (PMC11739848; doi:10.1002/cssc.202401471)
Supplement: Supplementary file 1 — Supporting Information [file CSSC-18-e202401471-s001.pdf]

ChemSusChem

Supporting Information

s-scheme3D/3D Bi^0/BiOBr/P Doped g-C3 N4 with Oxygen Vacancies (Ov) for Photodegradation of Pharmaceuticals: In-situ H_2O_2 Production and Plasmon Induced Stability

Mope E. Malefane,* Muthumuni Managa, Thabo T. I. Nkambule, and Alex T. Kuvarega*

Supporting Information

S-scheme 3D/3D Bi/BiOBr/P doped g-C_3N_4 with oxygen vacancies (Ov) for Photodegradation of Pharmaceuticals: In-situ H_2O_2 Production and Plasmon Induced Stability

Mope E. Malefane*, Muthumuni Managa, Thabo T. I. Nkambule, Alex T. Kuvarega*

Institute for Nanotechnology and Water Sustainability, College of Science, Engineering and Technology, University of South Africa, Florida, 1709, Johannesburg, South Africa

*Corresponding authors:

Mope E. Malefane, *University of South Africa, Florida, 1709,* Email: 62231855@mylife.unisa.ac.za

Muthumuni Managa, *University of South Africa, Florida, 1709,* Email: managme@unisa.ac.za

Thabo T. I. Nkambule, *University of South Africa, Florida, 1709,* Email: nkambtt@unisa.ac.za

Alex T. Kuvarega, *University of South Africa, Florida, 1709,* Tel: 0116709198, Email: kuvarat@unisa.ac.za

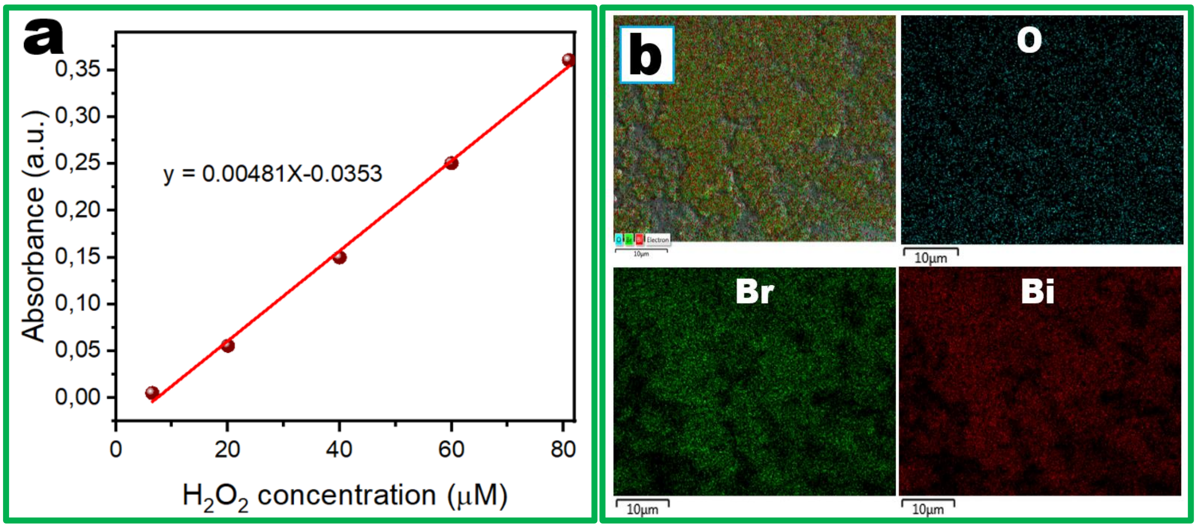

Fig. S1. (a) Calibration curve for H_2O_2 concentration and (b) EDX mapping of BOR.

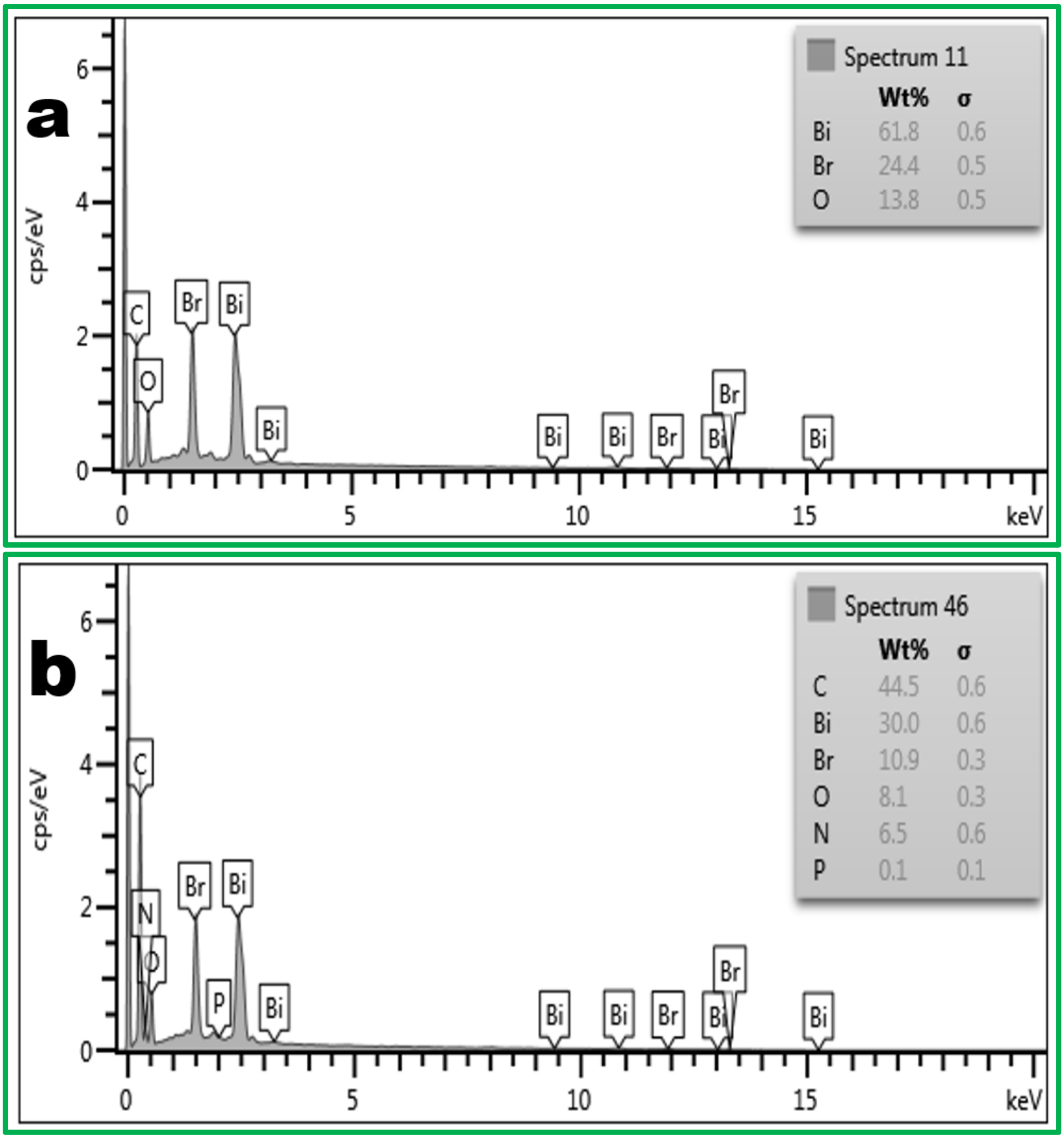

Fig. S2. EDX spectra of (a) BOR and (b) BORCNPO7.5.

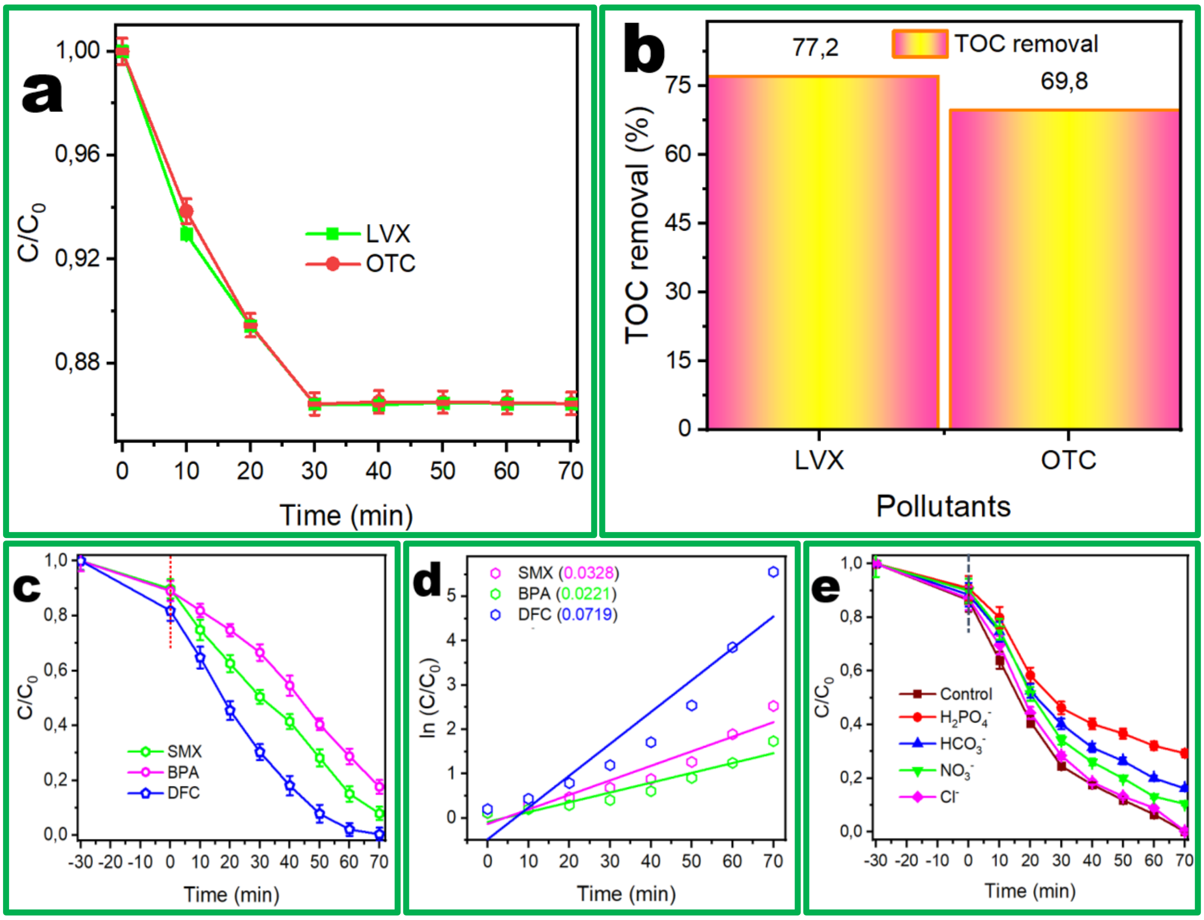

Fig. S3. (a) Adsorption and (b) TOC degradation efficiency by BORCNPO7.5. Degradation of other pollutants with BORCNPO7.5 (c) degradation curves, and (d) corresponding rate constants. (e) Effect of different anions on degradation of LVX by BORCNPO7.5.

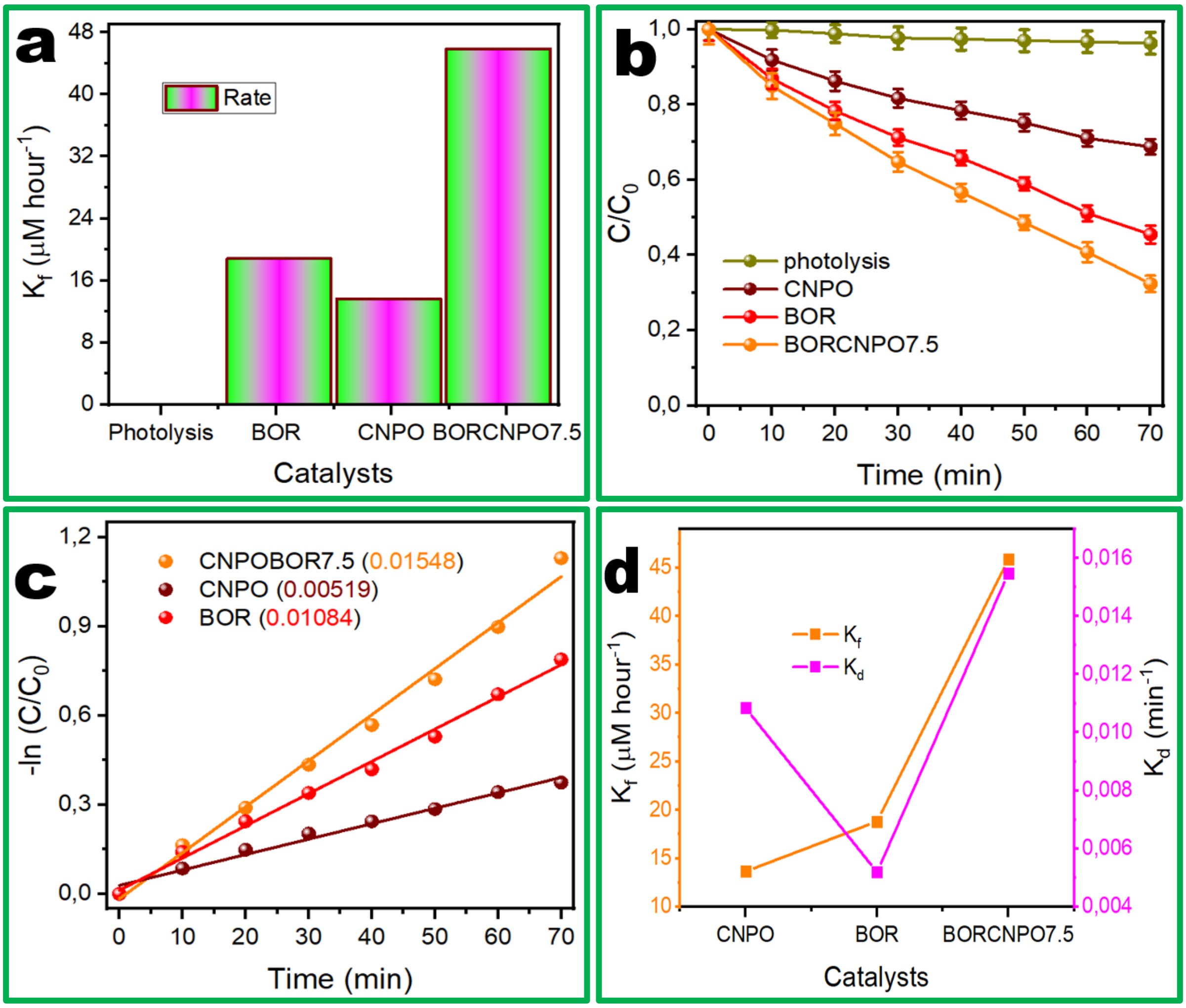

Fig. S4. (a) H_2O_2 formation rate, (b) decomposition graph, (c) decomposition rate of different catalysts, and (d) comparison of H_2O_2 formation and decomposition.

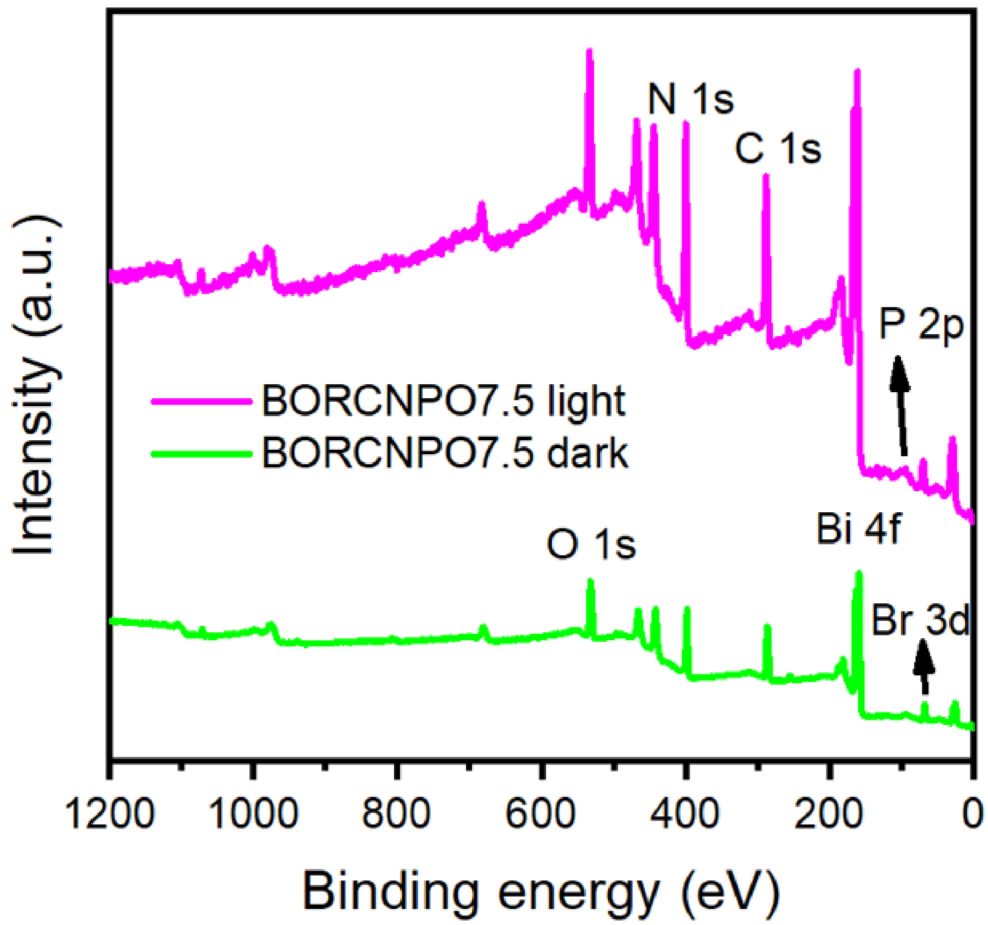

Fig. S5. XPS Survey spectra of BORCNPO7.5 in dark and under light.

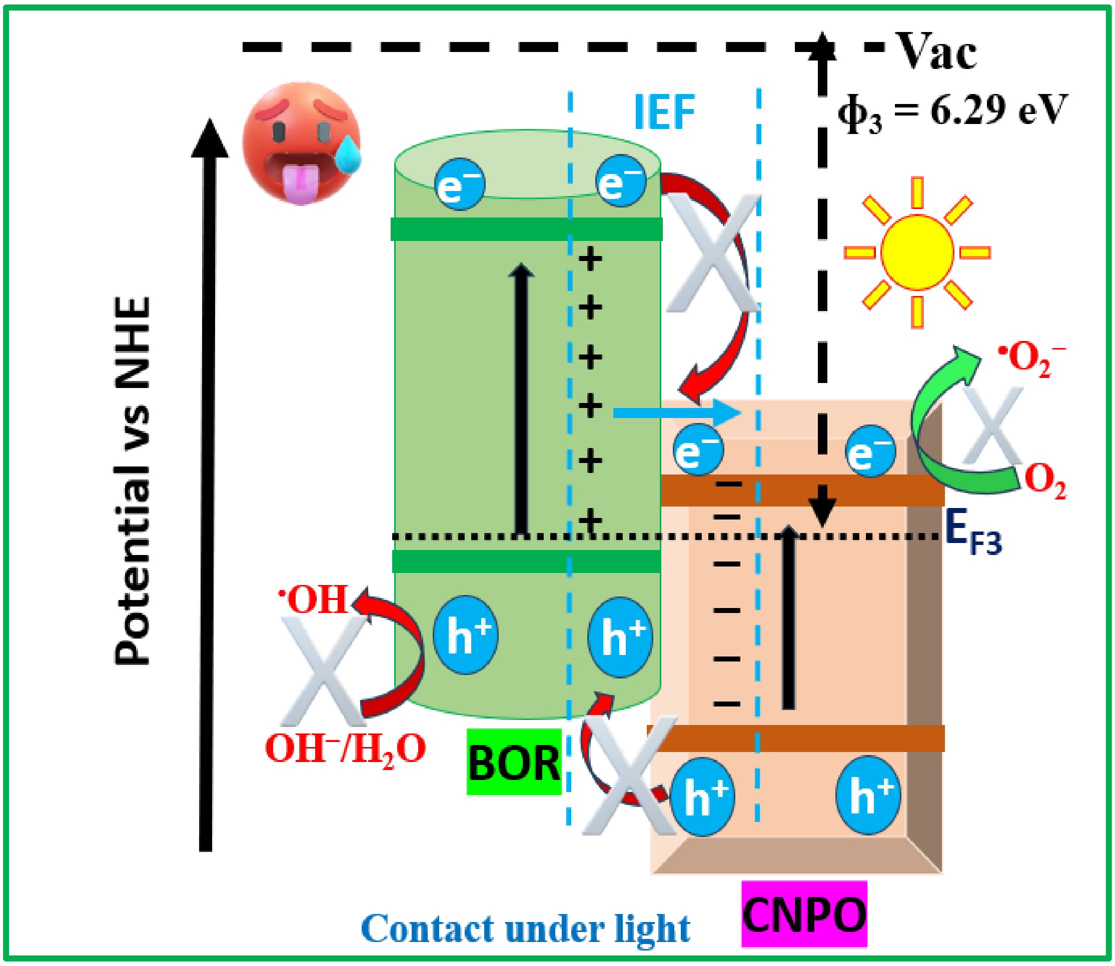

Fig. S6. Charge transfer for a type II heterojunction between BOR and CNPO.

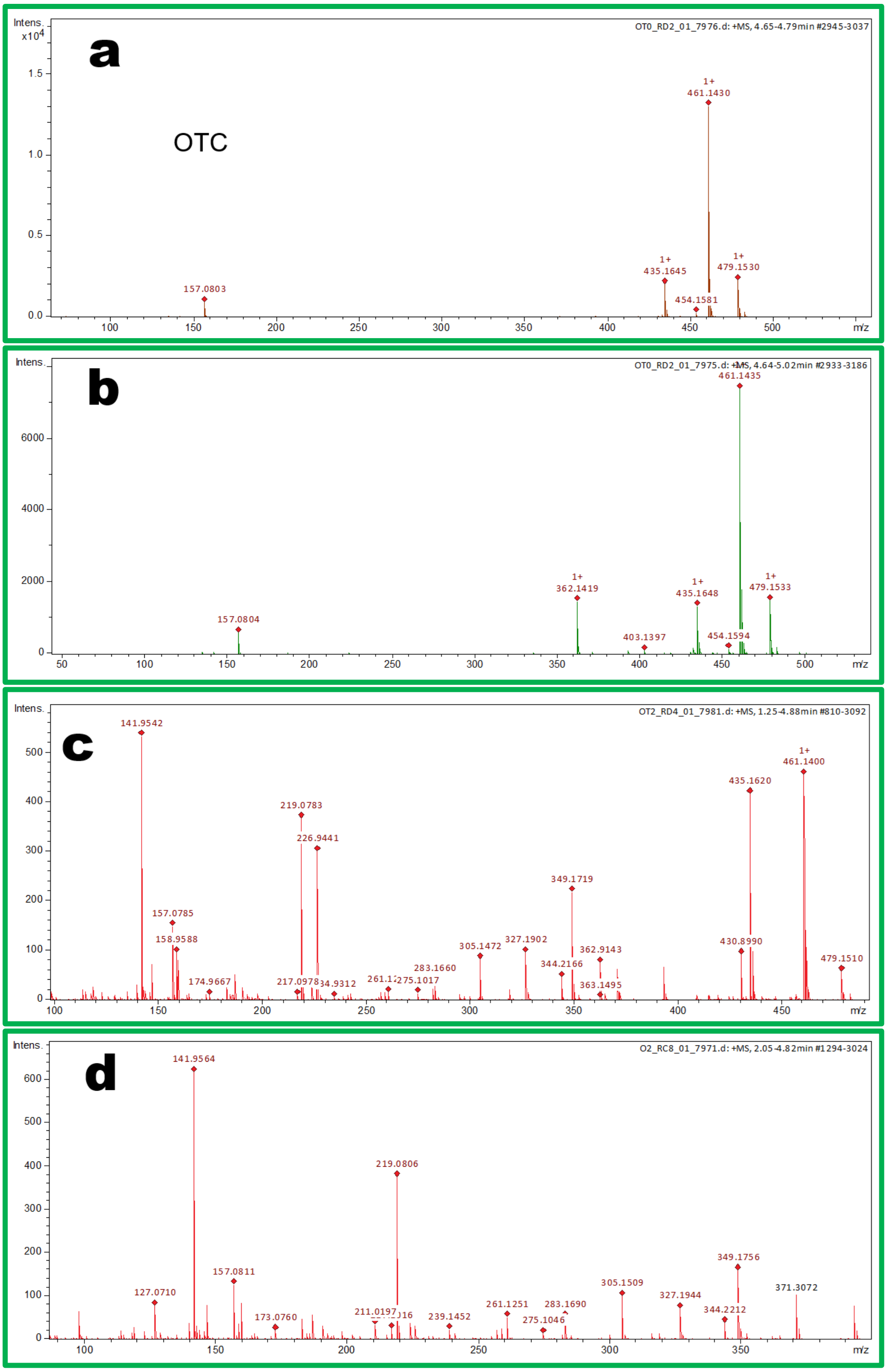

Fig. S7. QTOF-HPLC/MS spectra during degradation of OTC (a) 0 min, (b) 20 min, (c) 50 min, and (d) 70 min with BORCNPO7.5

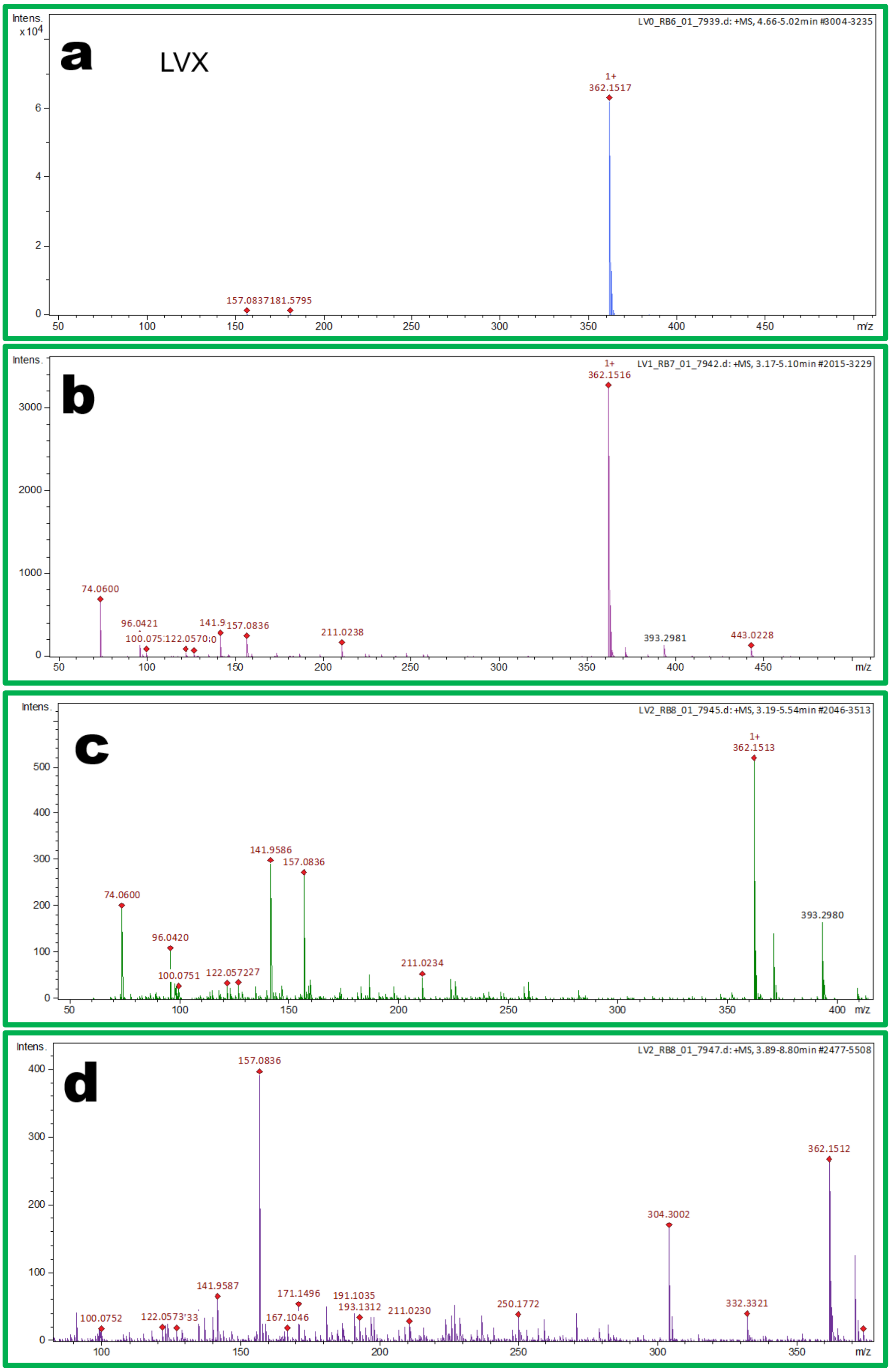

Fig. S8. QTOF-HPLC/MS spectra during degradation of LVX (a) 0 min, (b) 20 min, (c) 50 min, and (d) 70 min with BORCNPO7.5
